# A strategy of consistent X-ray and neutron double-difference pair distribution function analysis of nanoparticle dispersions

**DOI:** 10.1007/s00396-024-05333-z

**Published:** 2024-10-16

**Authors:** Sabrina L. J. Thomä, Joerg Neuefeind, Tristan G. A. Youngs, Mirijam Zobel

**Affiliations:** 1 Institute of Crystallography, RWTH Aachen University, Jägerstr. 17-19, 52066 Aachen, Germany; 2 Center for X-Ray Analytics, Empa – Swiss Federal Laboratories for Materials, Science and Technology, Überlandstrasse 129, CH-8600 Dübendorf, Switzerland; 3 Neutron Scattering Science Directorate, Oak Ridge National Laboratory, 1 Bethel Valley Road, Oak Ridge, TN 37831-6475 USA; 4 ISIS Neutron and Muon Source, STFC Rutherford Appleton Laboratory, Harwell Campus, Didcot, Oxfordshire OX11 0QX UK; 5 JCNS-3: Neutron Analytics for Energy Research, Forschungszentrum Jülich GmbH, Wilhelm-Johnen-Straße, 52428 Jülich, Germany

**Keywords:** Nanomaterials, Neutron pair distribution function (PDF), Double-difference PDF, NOMAD, NIMROD

## Abstract

It has been demonstrated that the X-ray pair distribution function (PDF) formalism allows for the identification of very small signal contributions in multi-component systems by the difference and double-difference PDF (dd-PDF) approach. Due to their stronger interaction with light elements compared to X-rays, neutrons are often beneficial or complementary for the characterization of modern materials. Here, it is demonstrated that the dd-PDF strategy previously developed for X-ray PDF data can successfully be applied to neutron PDF data despite much lower count rates compared to X-rays. The dd-PDF strategy was employed for the investigation of aqueous iron oxide nanoparticle (IONP) dispersions. At the Near and InterMediate Range Order Diffractometer (NIMROD) at ISIS, the IONPs could even be investigated in pure water, whereas at the Nanoscale Ordered Materials Diffractometer (NOMAD) at SNS, heavy water had to be used, but additional information could be retrieved from modelling the data of IONP powder in the dry state and with adsorbed (heavy) water. The simple and robust approach can easily be adapted for the use in other multicomponent systems, like heterogenous catalysts or battery systems.

**Graphical Abstract:**

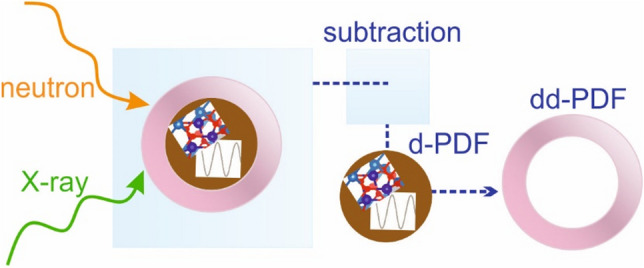

**Supplementary Information:**

The online version contains supplementary material available at 10.1007/s00396-024-05333-z.

## Introduction

In various multi-component functional materials, it is essential to detect light elements next to heavier elements. Prominent examples from the field of energy materials are hydrogen and carbon in fuel cells containing noble metal catalyst particles, or oxygen in multi-cation spinel and perovskite materials as used for electrodes. Neutrons are particularly sensitive to the light elements, while the interaction of X-rays becomes more dominant for heavier elements. Complementary studies using X-rays and neutrons are therefore frequently found [1, 2] and it was highly beneficial to have comparable data processing strategies at hand for complementary insight.

In contrast to conventional crystallography, the PDF formalism does not rely on long-range periodicity. Therefore, its use is particularly appropriate in the field of nanostructured materials lacking such long-range order. Examples include electrode materials [1, 3] and electrolytes [2, 4]. The PDF is proportional to a histogram of all interatomic distances within a sample and can be obtained by Fourier transformation (FT) of diffraction data. With the strongly increasing brilliance of synchrotrons and new detector technologies for high-energy X-rays, the detection of very small signals and scattering contributions became possible. In addition to many in situ studies on nanoparticle formation [5–7], for instance, pharmaceutical nanoparticles of circa 17 nm diameter were reliably detected in aqueous dispersion down to a concentration of 0.25 wt% [8]. We have shown the ubiquitous existence of solvation shells around < 10 nm nanoparticles in alcohols [9] and during self-assembly of truncated iron oxide cubes in organic solvents [10], detecting scattering contributions as small as 0.2% of the total scattering signal [11].

Neutron sources enable neutron PDF data collection with decent time resolution and flux, and we refer to Dove and Li’s recent review on this topic [12]. The historical strength of neutron total scattering in combination with isotopic substitution lies in its capability to study the solvation structure of ions [13]. Nowadays, solvation structures are, e.g., elucidated in new unconventional solvents like deep eutectic systems [14], and with the first oxygen isotope substitution experiment on a solute, the oxyanion solvation structure in saturated potassium nitrate has been elucidated [15].

In analogy to the just mentioned first-order difference analysis of neutron diffraction isotopic substitution experiments, X-ray PDF studies frequently employ difference- or double-difference-PDFs (d-PDF, dd-PDF)—aside from multi-phase refinements [16]—to access very small structural differences between samples and datasets [5–10]. In the case of nanoparticle dispersions, these d- and dd-PDFs are gained by subtracting the scattering of the bulk solvent and the nanoparticle powder from the dispersion data. In order to merge insight from X-ray and neutron PDF in a well-comprehensible and straightforward manner, analogous data analysis strategies are beneficial. In the case of neutron total scattering, to the best of our knowledge, only studies investigating adsorbed species such as gas–solid interfaces [17, 18], as well as surface-bound water on wet powders [19], exist. Here, we want to bridge the gap to the investigation of nanoparticle dispersions complementary to X-ray PDF. Because of the rising importance of neutron PDF for functional nanostructured materials [16, 19–21], in this article, we show how to expediently calculate d- and dd-PDFs from neutron PDF data collected at two state-of-the-art neutron PDF instruments, the Near and InterMediate Range Order Diffractometer (NIMROD) [22] at ISIS and the Nanoscale Ordered Materials Diffractometer (NOMAD) [23] at the Neutron Spallation Source (SNS), based on the X-ray dd-PDF approach [9, 24]. We demonstrate this strategy for 100 mg/mL iron oxide nanoparticles (IONPs) dispersed in (heavy) water using (heavy) water as well as the dry powders as background. This study shows the high sensitivity of modern state-of-the-art neutron PDF instruments and proves that dd-PDF studies are likewise possible for X-ray data despite the lower count rates. The simple, yet powerful strategy, can easily be adapted for the use in other multicomponent, nanostructured systems, such as heterogeneous catalysts or battery materials.

## Materials and methods

In order to conduct a dd-PDF study, a full set of data of the nanoparticle dispersion and all the respective components of the dispersion are needed. Namely, these are the nanoparticle dispersion itself, the pure dispersant (here: water/heavy water), and the dry nanocrystalline powder. Two neutron total scattering experiments were carried out and the experimental details as well as the strategy for dd-PDF calculation are described below. Detailed information about the data reduction from the raw data to the here presented data on the normalized or absolute scale, performed in collaboration with the beamline scientists, can be found in the Supporting Information in Sect. 1. Synthesis and preparation of the samples are reported in Supporting Information in Sect. 2.

### dd-PDF experiment at NIMROD at ISIS

Neutron diffraction experiments were performed at the NIMROD TOF total scattering instrument [22] at the ISIS Neutron and Muon Source of STFC Rutherford Appleton Laboratory. The samples were measured in null-scattering TiZr alloy flat plate cans. Thereby, powder samples were measured in cells for a sample thickness of 2 mm, and about 500–600 mg of powder was needed to fill those cans. The more incoherently scattering IONP dispersions and liquid backgrounds (D_2_O, H_2_:O:D_2_O mixture (50:50), and H_2_O) were measured in cells for a sample thickness of 1 mm. IONP dispersions in D_2_O, H_2_:O:D_2_O mixture (50:50), and H_2_O with a concentration of 100 mg/mL were prepared on-site. Data over the full *Q*-range of the instrument from 0.02 to 50 Å^−1^ was acquired for 4 h on each sample, except for D_2_O, which was measured for 8 h. Most measurement time was spent on D_2_O, since the likelihood of obtaining reliable double-difference correlation functions was considered to be highest.

### dd-PDF experiment at NOMAD at SNS

Neutron TOF total scattering measurements were conducted at NOMAD [23] at SNS of the Oak Ridge National Laboratory (ORNL). All samples were measured in 3-mm-outer-diameter quartz glass capillaries to an accelerator proton charge of 12–16C, which corresponds to 2.5 to 3 h of measurement time at full power. The samples have been prepared in the home laboratory, filled in the quartz glass capillaries provided by ORNL, and then sent back for measurements (see also Supporting Information Sect. 2.2).

### dd-PDF calculation

The dd-PDFs were calculated from the PDFs on normalized (NOMAD) or absolute scale (NIMROD), as received after the data reduction, in the software environment Igor Pro 8 from WaveMetrics according to Eqs. 1 and 2:

1 $$d-PDF{= }{^\prime}PDF nanoparticle dispersio{n}{^\prime} {- }{^\prime}PDF dispersant{^\prime}$$

2 $$dd-PDF=d-PDF {- x\bullet }{^\prime}PDF dry powder{^\prime}$$

From Eq. 2, it is obvious that the PDF of the dry nanocrystalline powder was scaled to the d-PDF by applying the scale factor $x$. The scaling was conducted such that distance correlations in the *r*-range $>$ 7 Å (NIMROD, $>$ 15 Å NOMAD) match in intensity. This scaling is needed and justified by the fact that the nanoparticle contribution is naturally a lot smaller in the nanoparticle dispersion than in the dry powder (most of the atoms in the dispersion are in the water molecules). The scale factors *x* for the investigated samples are listed in Table 1.

**Table 1:** Scale factors *x* for dd-PDF calculation for respective samples

Sample	Scale factor *x*
IONP-cit-H_2_O	0.030
IONP-cit-H_2_O-D_2_O	0.030
IONP-cit-D_2_O	0.015
IONP-phos-H_2_O	0.035
IONP-phos-H_2_O-D_2_O	0.035
IONP-phos-D_2_O	0.025
IONP-cit-D_2_O NOMAD	0.021

## Results and discussion

Neutron total scattering data of aqueous IONP dispersions, (heavy) water dispersants, and dry IONP powder was acquired at two instruments, namely NIMROD [22] at ISIS and NOMAD [23] at SNS. The dd-PDF strategy developed for X-ray data [9, 24] was adapted and applied to retrieve double-difference-signals for data sets acquired at both instruments and the data of both instruments with their respective advantages and limitations is compared within this article. Due to the higher sensitivity of neutrons towards light scatterers (C, H, and O) and the strength of neutron scattering in hydration studies [14, 15], initially, the aim of these experiments was to gain more detailed insight into the hydration shells of these IONP-based on the X-ray dd-PDF signal obtained by Thomä et al. [25]. During subsequent work [24], it was discovered that the previously obtained dd-PDF signal was misinterpreted and that these IONP dispersions are lacking hydration shells according to X-ray PDF data. The X-ray dd-PDF extraction is shown in the study by Thomä and Zobel [24] revealing dd-PDF signals close to zero. Accordingly, no strong dd-PDF signal is expected for these samples and they are admittedly not the perfect target system for a proof-of-principal study. Nevertheless, the data show that d- and dd-PDF studies are possibly likewise to X-ray data and demonstrate the great sensitivity and capability of modern state-of-the art neutron total scattering instruments. Therefore, the data of both instruments with their respective advantages and limitations are compared within this article.

Different communities have adopted slightly different conventions in the presentation of the pair distribution function. However, the physical content of these different presentations is of course the same. They all provide information about interatomic distances, average coordination numbers, and mean square displacements [26]. The derivations and conversions have been set out and discussed in detail in the literature [26–28]. We provide a summary thereof in the Supporting Information in Sect. 3, including a derivation of the magnetic PDF (mPDF), since the investigated IONPs contain also a mPDF contribution.

For a comprehensible summary for beginners on the initial data treatment to deduce the neutron PDFs from the actual raw data, we refer the interested reader to Dove and Li’s review [12]. It involves several necessary steps, such as (i) corrections for incoherent scattering, absorption, and attenuation of the beam from other sources than the sample; (ii) converting TOF data from function of velocity to function of *Q*; (iii) normalization to calibrate detectors against each other and subtraction of self-scattering term; and (iv) correction for inelastic effects. The users are guided through the process of how to obtain the corrected neutron total scattering data by the respective support scientists at the neutron facility with the facility-specific software product and in the end obtain the data either on a normalized or absolute scale. Here, we present the dd-PDF strategy based on these absolute or normalized neutron PDFs. For the data acquired at NOMAD at SNS the dimensionless total scattering structure function as well as the real-space distribution function PDF(*r*), whereas for NIMROD data the interference differential cross section *F(Q)*, in units of *barns sr*^*−1*^* atom*^*−1*^ and the differential correlation function *D(r)* in units of *barns atom Å*^*−2*^ were received. The relationship between these functions is given in Eqs. 3 and 4, where *c*_i_ is the weight factor of atom *i* and ${b}_{i}$ is the respective coherent neutron scattering length, showing that the presented functions differ by constant factors:

3 $$F\left(Q\right)= \sum {({c}_{i}{b}_{i})}^{2}\bullet \left[S\left(Q\right)-1\right]$$

4 $$D(r)= 4\pi \rho \sum {\left({c}_{i}{b}_{i}\right)}^{2}\bullet PDF(r)$$

### Experiment at NIMROD

**Fig. 1:**
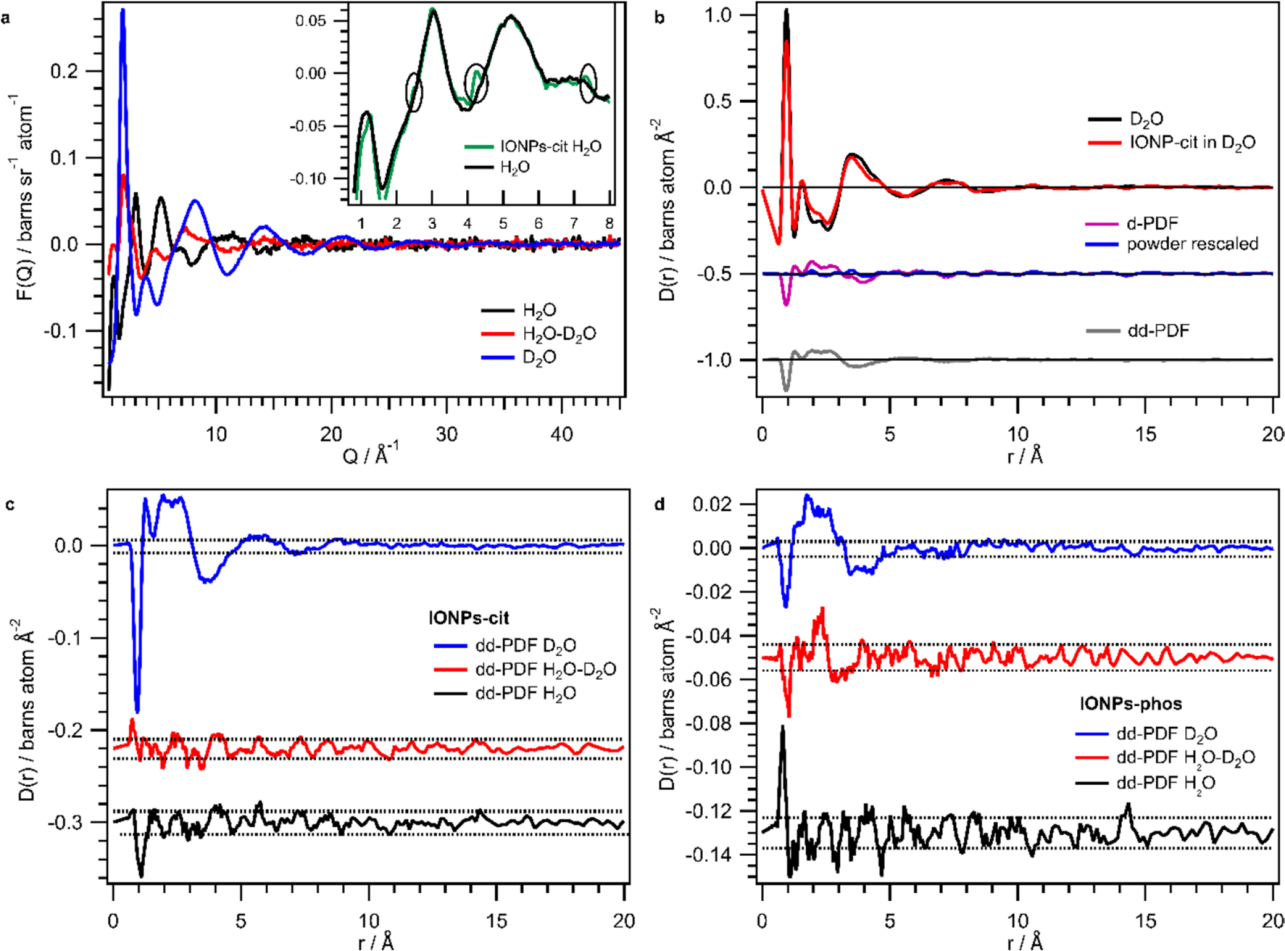
*F(Q)* and *D(r)* data of IONP dispersions acquired at NIMROD. **a** *F(Q)* of the signals measured on the pure solvents, namely H_2_O (black), D_2_O (blue) and 50:50 H_2_O:D_2_O mixture (red). In the inset, a zoom into the low *Q*-region for *F(Q)* of H_2_O and IONPs-cit in H_2_O is given, highlighting the IONP signal in circles. **b** Derivation of the double-difference signal (grey) from *D(r)* data, by first subtraction of D_2_O (black) from the dispersion data (red) and subsequent subtraction of the rescaled powder signal (blue) from the difference signal (violet). **c** Double-difference-signals of *D(r)* for IONP-cit samples in D_2_O (blue), H_2_O (black), and 50:50 mixture (red). For illustrative purposes, double-difference-signals for D_2_O-H_2_O and H_2_O are in offset. **d** Double-difference-signals of *D(r)* for IONP-phos dispersion in H_2_O, D_2_O, and D_2_O-H_2_O with D_2_O-H_2_O and H_2_O signal in offset

At NIMROD aqueous dispersions of IONPs (H_2_O, D_2_O, H_2_O: D_2_O (50:50)) capped with two different ligands, citrate and phosphocholine (sample names given are IONPs-cit and IONPs-phos) were investigated. As mentioned in the “Materials and methods” section, measurement of the respective dry powders and liquid background is needed additionally for the dd-PDF extraction. Figure 1 shows these data and visualizes how the double-difference strategy was applied.

In Fig. 1a, the *F(Q)* of pure solvents (H_2_O, D_2_O, and a 50:50 mixture of the two), which is the background in the dispersions, is shown. With increasing H content and thus incoherent scattering contribution, the noise in the data increases; nevertheless, an acceptable data quality was obtained for all three liquid samples. Further, the inset shows that even for the quite noisy water data, Bragg scattering from the IONPs in a 100 mg/mL dispersion is clearly discernible from noise showcasing the high sensitivity of this neutron total scattering instrument. Figure 1b shows the dd-PDF extraction as described in the “Materials and methods” section. First, a d-PDF (violet) from the dispersion minus the pure solvent is obtained to which in a second step, the powder data (blue) are rescaled. This scaling will be shown in more detail later in direct comparison to the data from NOMAD. Finally, a small dd-PDF is extracted by subtracting the rescaled powder *D(r)* from the d-PDF. Figure 1c and d display double-difference-signals of IONP-cit dispersions and IONP-phos dispersions in comparison. It is evident that likewise as for the data in reciprocal space, the noise in the double-difference-signals increases with increasing hydrogen content. The noise level was estimated at medium *r* range ($>$ 15 Å), where no contribution from a potential hydration shell is expected anymore [9]. For all samples, the dd-PDF contains barely a signal above this noise level. Only the OH/OD distance (0.96 Å) is prominently visible, except in IONP-cit H_2_O-D_2_O which is only slightly above noise.

In this experiment, the acquired powder data was only used for the subtraction of the nanoparticle contribution. No detailed investigation and modelling of the powder data are provided, since the nanoparticle signal in *D(r)* was dampened too strongly (cf. Supporting Information Sect. 4). More information about the total composition of the powders, important for the processing of the neutron PDF data, and the NIMROD powder PDF data themselves can be found in the Supporting Information in Sect. 4 and in the first author’s dissertation.

### Experiment at NOMAD

**Fig. 2:**
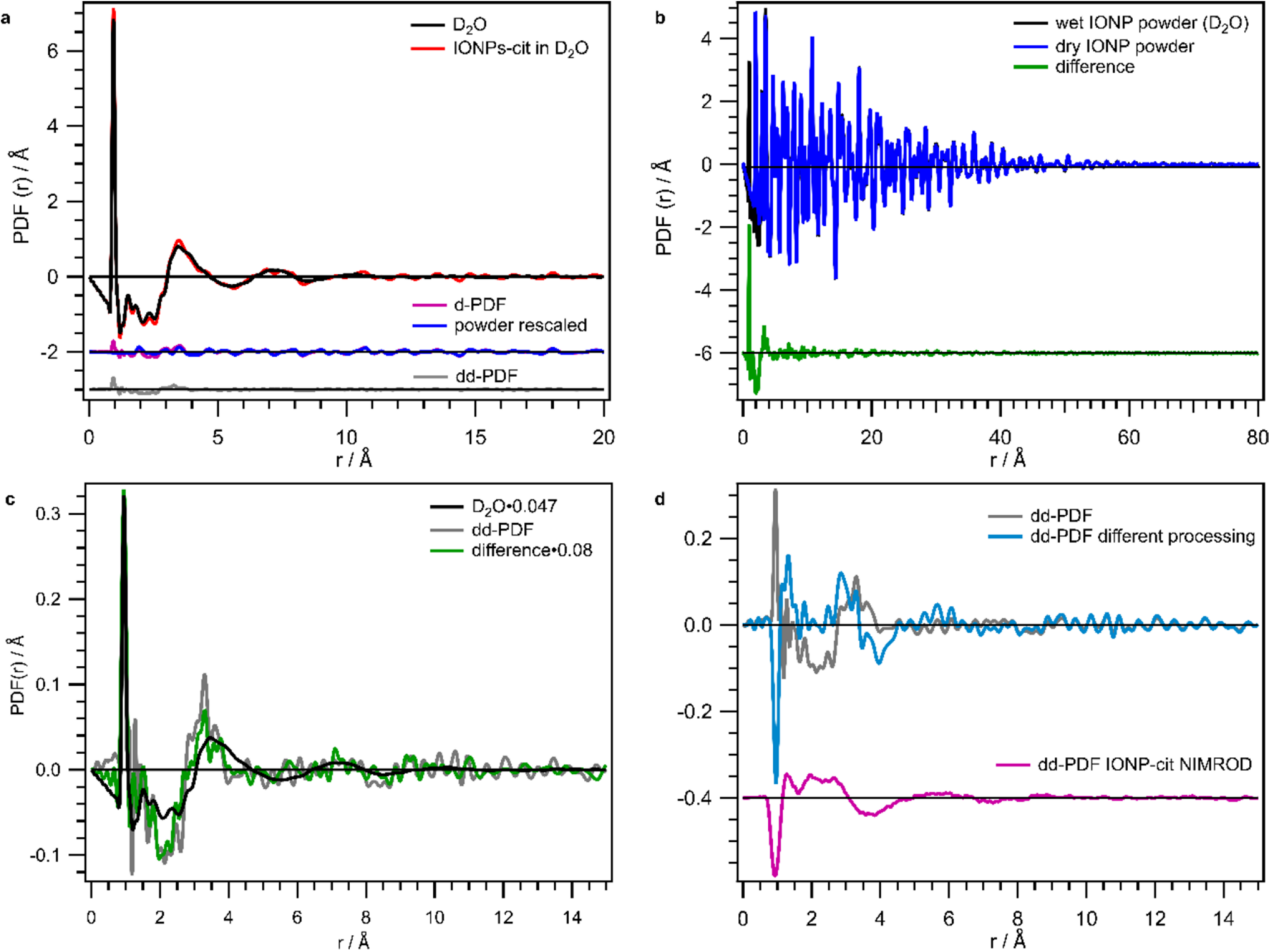
*S*(*Q*)-1 and *PDF(r)* data of IONP dispersions acquired at NOMAD. **a** Derivation of the dd-PDF (grey) of IONPs-cit dispersion (red) by subtraction of D_2_O background (black) and subsequent scaling and subtraction of powder PDF (blue) from the d-PDF (violet) for NOMAD data. **b** *PDF(r)* of a D_2_O wet IONP powder (black) is compared to a dry IONP powder (blue), with their difference (green) in offset. **c** The dd-PDF from **a** (grey) is shown in comparison to the difference (green) of wet and dry powder from **b** and the rescaled PDF of D_2_O (black). **d** The comparison of the dd-PDF from **a** (grey) and this dd-PDF derived with different processing (blue; no normalization) is amended in offset by the dd-PDF of IONP-cit dispersions derived from *D*(*r*) acquired at NIMROD (pink; offset − 0.4). The *y*-axis is not labelled, since *D*(*r*) and *PDF(r)* are presented in the same plot

At NOMAD, the dd-PDF experiment was conducted with an IONP-cit dispersion in heavy water at a concentration of 100 mg/mL. The experiment was also attempted for the IONPs dissolved in a 50:50 mixture of H_2_O:D_2_O, but failed since no meaningful data were obtained. Additionally, at NOMAD, one IONP-cit sample has been investigated as nominally “dry” powder (equilibrated at a relative humidity (RH) of 11% see Supporting Information Sect. 2.2.) and as a powder with adsorbed water and with adsorbed heavy water (both equilibrated at a RH of 96% see Supporting Information Sect. 2.2.). In Fig. 2, the data acquired for the IONP heavy water dispersion and as resulting from the applied dd-PDF strategy are visualized.

In Fig. 2a, the dd-PDF retrieval for the IONP-cit heavy water dispersion likewise to Fig. 1b is depicted. The obtained dd-PDF (grey line, offset − 3) is obtained, which is expectedly small. Since a hydration shell in the IONP dispersion could be similar to an adsorbed water layer on IONP powder, the obtained dd-PDF should be compared to the difference between wet and dry powder (see Fig. 2b). In Fig. 2c, this obtained difference (green) rescaled by 0.08 as well as the dd-PDF (grey) from IONP-cit dispersion in heavy water are compared to the PDF curve of bulk D_2_O rescaled by 0.047 (black). Both the dd-PDF and the difference between wet and dry powder carry more noise, such as a noise ripple on top of the first nearest neighbor distance at $\sim$ 4 Å, in comparison to bulk D_2_O. Additionally, this first nearest neighbor correlation seems to be a bit narrower and a bit shifted to lower distances in comparison to bulk heavy water. This could be interpreted such that an adsorbed water layer, on wet powder or in dispersion, possesses a bit higher density than bulk heavy water.

Yet, it should be mentioned that slight differences in processing changed the signal shown in Fig. 2d. The dd-PDF from IONP-cit NOMAD data received, when subtracting the unnormalized data from each other with no Fourier filter applied (blue in Fig. 2d), resulted in a very similar signal to the one obtained at NIMROD (pink signal in offset in Fig. 2d). These dd-PDF signals seem to be the negative analogue of the PDF of heavy water (cf. Figure 2c). We will come back to the discussion of the dd-PDF signals at a later point, when the two experiments are compared.

**Fig. 3:**
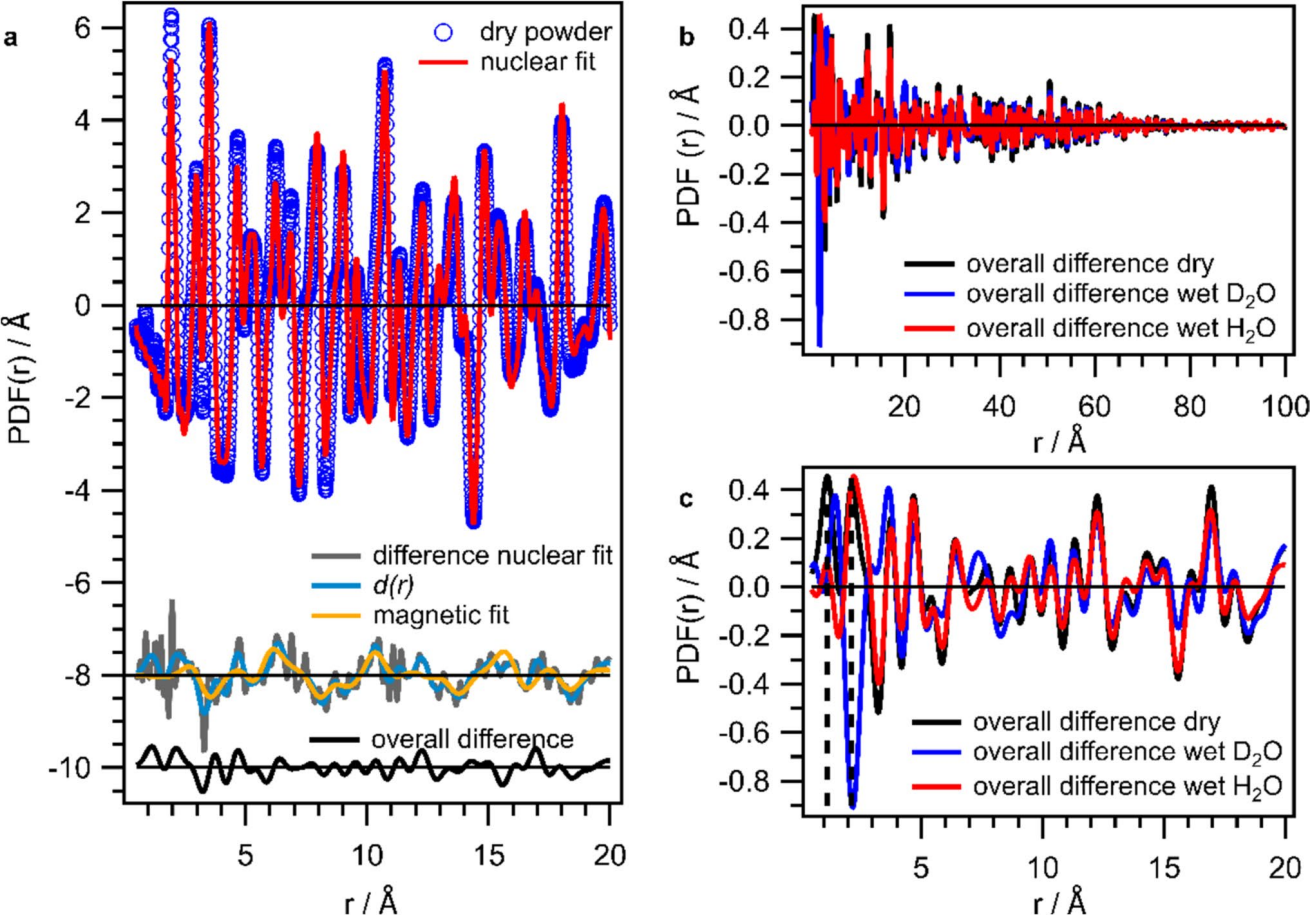
Modelling of *PDF(r)* data of IONP powders with its results. **a** Nominally dry IONP powder with its fit for nuclear and magnetic contribution over an *r*-range up to 20 Å. Measured *PDF(r)* data are given in blue, its nuclear fit in red, and the resulting difference in grey in offset at − 8. The resulting difference was smoothed to obtain *d*(*r*) (light blue in offset, − 8) and fitted with a magnetic model (yellow in offset, − 8). The overall difference is given in black in offset − 10. **b** The resulting overall differences after modelling the atomic as well as magnetic contribution of the total neutron *PDF(r)* for all three powder samples for the full* r*-range up to 100 Å. **c** The overall differences for all three evaluated powders are shown in comparison for the short-range order *r*-range up to 20 Å. Dashed lines indicate distances at 1.1 Å and 2.1 Å

However, the PDF of the powder (wet and dry) cannot only be used for this comparison. With the investigation of “wet” powders likewise to the study by Wang et al. [19] and complementary to a dd-PDF study on dispersions, one can elucidate the full hydration process from a dry particle over particle with few water layers on top, up to particles fully dispersed in (heavy) water). In order to retrieve information about the capping agent citrate and to check if surface hydroxyl groups are present in the “nominally” dry powders, the *PDF(r)* data of “nominally” dry and the two wet powders were modelled. Figure 3 visualizes and summarizes which additional information can be gained from the evaluation of the powders.

In Fig. 3a, the modelling of the *PDF(r)* data of the dry IONP powder is shown exemplarily for the short *r*-range up to 20 Å. The data for the *r-*range up to the full decline of the PDF (80 Å), as well as the data of the other two powders, all fit values for the atomic and magnetic neutron PDF refinement, complementary X-ray PDF data, and further information can be found in the Supporting Information Sect. 5. First, the atomic structure was fitted with a P4_3_2_1_2 phase [29] for all three powders. The IONPS are hypothesized to consist of a Fe-richer core and a more oxidized outer region with a structurally coherent transition similar to the yet larger IONPs investigated by Andersen et al. [21], also based on X-ray PDF data. No peak for an OH distance was detected in the dry powder revealing no obvious contribution of surface OH. For the wet powders, an additional phase similar to Wang et al. [19] and Plekhanov et al. [30] was added to account for the positive OD and negative OH peaks exhibited at 0.95 Å and 1.01 Å. However, here no information about the water content can be retrieved from the peak area of the OH/OD peak as discussed by Wang et al. [19], because only one wet powder each (D_2_O/H_2_O) was measured.

As expected for a magnetic material, the difference curves from the neutron *PDF(r)* fits of the atomic structure still exhibit a broad oscillatory signal (see Fig. 3a signals in offset at − 8). This difference signal is smoothed to obtain *d(r)*, the unnormalized mPDF (cf. Supporting Information Sect. 3 Eq. 14) and it was modelled with the ferrimagnetic structure of IONPs also used by Andersen et al. [21], similar to bulk magnetite/maghemite.

In Fig. 3b and c, the resulting overall difference curves after modelling both, the crystalline atomic part and the magnetic contribution, are shown. The three difference curves, which apparently in turn are dd-PDFs, for the *r*-range up to 100 Å in Fig. 3b, show that the noise level is *r*-dependent and decreasing for higher *r*. This is related to the fact that PDF data collected on NOMAD does possess* r*-dependent instrumental resolution functions and therefore fits performed over large* r*-ranges ($>$ 30 Å, here 100 Å) suffer in quality [21, 23]. Nevertheless, it can be claimed that the unusually high residual curve for the short-range up to 20 Å (see Fig. 3c) is related to the contribution of the organic ligand and the adsorbed (heavy) water, which is not described by the crystal structure and the magnetic model (cf. Supporting Information Sect. 5.4). The peak at very low *r* of 1.10 Å (first dashed line) could relate to the missing normalization to the magnetic form factor of *d(r)*. The second leftover distance correlation appears at ca 2.10 Å is positive in dry and H_2_O wet powder and negative in D_2_O wet powder. We attribute it to be a leftover of the first Fe–O distance correlation to which different Fe–O distances (1.88–2.13 Å) within the unit cell contribute [29]. This distance has been underestimated a lot by the nuclear fit in Fig. 3a (see also Supporting Information Sect. 5.4). Moreover, distances of adsorbed water with surface Fe would also appear in this region of 1.9–2.3 Å, depending on surface termination (facet) and mode of adsorption (molecular/dissociative) [31]. Further, by water adsorption on iron oxide, surface Fe–O distances can be altered, since the surface ions are pulled out of the surface upon water adsorption (16–24 ppm) [31]. Thus, we hypothesize that this leftover peak at 2.1 Å is associated with (heavy) water adsorption for the reasons given above, matching the observation of a peak of opposite sign for D_2_O. For *r* > 3 Å, the three residual curves are all fairly similar, yet above the noise level, suggesting that there is some undescribed contribution of the organic ligand causing these mismatches.

## Comparison of the two experiments: respective advantages and limitations

**Fig. 4:**
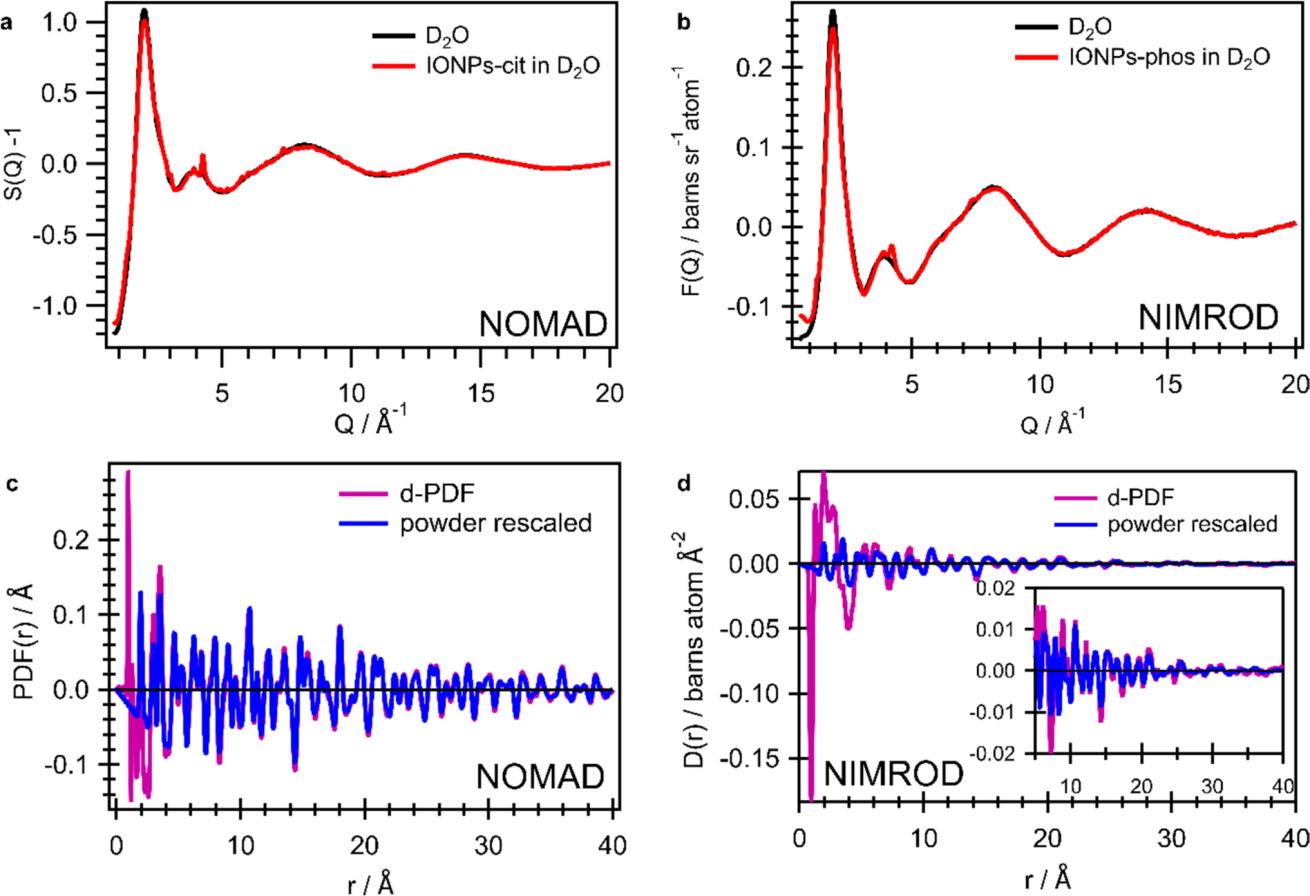
Total scattering data of an IONP dispersion acquired at NOMAD and NIMROD in comparison. Total scattering data in reciprocal space for the IONP dispersion and D_2_O dispersant acquired at **a** NOMAD and **b** NIMROD. Powder PDF rescaled to the d-PDF for the second step of the dd-PDF extraction in **c** for NOMAD data and **d** for NIMROD data

We now want to compare the data of the two experiments. In Fig. 4a and b, the obtained reciprocal space data of one IONP heavy water dispersion in comparison to pure D_2_O signal, which is the background, are shown for the two experiments for (a) NOMAD and (b) NIMROD. The Bragg peaks are nicely and clearly visible on top of the water signal up to *Q* values of $\sim$ 12 Å^−1^ for NOMAD and 7.5 Å^−1^ for NIMROD. The apparently smaller Bragg scattering in the NIMROD data is for sure partly caused by the worse *Q*-resolution of the instrument (cf. width of Bragg peak at 4.5 Å^−1^). Since the resolutions are 0.7% *dQ/Q* for NOMAD vs. 3% *dQ/Q* for NIMROD, the difference in intensity seems quite high. The dispersions were all measured with a similar concentration of approximately 100 mg/mL (except IONP-phos in H_2_O was a bit lower concentration; cf. Supporting Information Sect. 2.3). Further, all IONPs were of similar size and possess similar content of organics. The experiment at NOMAD was a mail-in experiment, and hence, the dispersion was not prepared directly before the measurement which is different from the one at NIMROD. Even though no precipitation and phase separation were visible in the capillaries, weak agglomeration can not be ruled out as a source of higher Bragg scattering.

Figure 4c and d depict the second step of the dd-PDF extraction in real space, namely the scaling of the powder PDF to the d-PDF (difference between dispersion and dispersant). After subtraction of the huge background of the pure dispersant (cf. Figures 1b and 2a), the small contribution of the IONP powder becomes clearly visible and the powder PDF can be nicely scaled to the d-PDFs for both instruments. Thus, this illustrates the very high sensitivity of the two state-of-the-art neutron instruments in Fig. 4 in (c) for NOMAD and (d) for NIMROD. The agreement between d-PDF and rescaled powder PDF is nearly perfect in the case of NOMAD above 10 Å, whereas the deviations are a bit more apparent in the case of NIMROD data. In this case, the scaling is impeded by the fact that the powder PDFs are already strongly dampened beyond 10 Å, which is a result of the *Q*-resolution.

Thanks to the good *Q* resolution at NOMAD (wet) powders can be refined similar to the study by Wang et al. [19] to bridge the gap from powders to dispersions, as discussed in Fig. 3. By a study with varied (heavy) water content on the powders, the full hydration process could be elucidated. However, we are not able to obtain meaningful data for hydrogenated samples with NOMAD applying the usual data treatment optimized for high flux (full angular range), whereas at NIMROD, the IONPs could be investigated even in water with natural isotopic composition (cf. Figure 1). This is a great advantage, even though the study is limited to the short-range order, since H_2_O and D_2_O have different physicochemical properties. Modified hydrogen bond networks as induced by H–D exchange are expected to influence the hydration behavior, and consequently, we cannot expect that interaction in H_2_O can be fully transferred to D_2_O. H/D-isotope effects have, e.g., already been reported to affect the size of copolymer micelles [32], as well as the gold [33] and cadmium sulfide [34] nanoparticle formation. For cases where the assumption of H_2_O and D_2_O possessing the same structure is valid, measuring solutes (or nanoparticles) in H_2_O, D_2_O and a 50:50 mixture of the two opens the possibility for a second-order difference study [35].

Concluding, we want to take a look again at the extracted dd-PDFs in Fig. 1c and d, as well as in Fig. 2d. We already realized that the OD distance peak is positive for the dd-PDF extracted for the IONP-cit dispersion investigated at NOMAD, in contrast to the dd-PDFs for heavy water dispersion extracted from NIMROD data. This is of course due to the fact that the OD distance correlation is higher in the dispersion than in the D_2_O background in this case (cf. Figure 2b), in contrast to data taken at NIMROD (cf. Figure 1b). Intuitively, one could say that we expect rather the case of the NIMROD data that there are fewer OD distances in the dispersion compared to bulk water, since the water contribution itself is smaller. However, the exact number of OD distances here depends on many different factors, such as the coverage of the surface with ligand, which in turn depends on the exact particle size, shape, and amount of ligand, all very difficult to absolutely control and determine. Even though all investigated IONP powders were similar in size and ligand content, this could be an explanation for reversed peak intensities (cf. also Fig. 1c and d dd-PDFs of H_2_O-D_2_O and H_2_O). In any case, the difference in this peak height is expected to be very small and the absolute or normalized processing of these data, containing the question of how much free D_2_O (H_2_O) is there in concentrated IONP dispersions, becomes to some extent arbitrary. This is due to the fact that the data processing and corrections include several levels of uncertainty, such as the imprecisely known effect of inelasticity, correction for the self-scattering term being largest for light elements and large angels, and merging data from different detector banks. All mentioned factors are far from being trivial [12]. The retrieved grey dd-PDF signal of IONP-cit dispersion at NOMAD seems reasonable, and for the other two cases presented in Fig. 2d, it rather seems like an over-subtraction of the heavy water background. However, we do not certainly know if this is the case and cannot completely exclude incomplete subtraction of the D_2_O background in the case of the IONP-cit NOMAD data either.

Nevertheless, we believe that the data show the great sensitivity, capability, and limitations of neutron state-of-the-art instruments for d- and dd-PDF studies and refer the reader again to Fig. 4.

## Conclusion

The dd-PDF strategy previously developed for X-ray PDF data of nanoparticle dispersions was successfully applied to neutron total scattering data acquired at two different neutron PDF instruments. Bragg scattering from IONPs of about 6–7 nm was detected at a concentration of 100 mg/mL in aqueous dispersions. A double-difference signal from the aqueous dispersions could be obtained after subtracting the pure solvent signal and the rescaled powder data for both instruments. The powder PDF data can be nicely scaled to difference signals between dispersions and pure solvents, which shows the great capability of state-of-the-art neutron total scattering diffractometers. The great advantage of the NIMROD instrument was that even IONPs in pure H_2_O could be evaluated, while at NOMAD, additional information can be gained by studying powders with varied hydration levels likewise to the study by Wang et al. [19], which will help to understand the full process of hydration. We conjecture that structural analysis of various nanostructured materials involving fuel cells or multicomponent battery systems can benefit from this double-difference approach in PDF data analysis, which is likewise possible for both X-ray and neutron PDF data. With upcoming higher brilliance at diffractometers such as DREAM [36] at ESS or brilliance upgrades at ISIS within the Endeavor program, we expect that the detection of solvation phenomena around nanoparticles in colloidal dispersions will hopefully become possible in the near future with the herein presented approach.

## Supplementary Information

Below is the link to the electronic supplementary material.

Supplementary file1 (DOCX 803 KB)

## Data Availability

Data was taken at ISIS Neutron and Muon Source (STFC Rutherford Appleton Laboratory) from proposal RB1920266 as well as at the Spallation Neutron Source (Oakridge National Laboratory) from proposal 25873.1. The experimental raw data is stored at the respective facilities. The raw data can be acessed upon reasonable request from the corresponding author.
