# Supplementary material for: A strategy of consistent X-ray and neutron double-difference pair distribution function analysis of nanoparticle dispersions
Source: Colloid Polym Sci. 2024 Oct 16;303(9):1843–52. doi: 10.1007/s00396-024-05333-z (PMC12443920; doi:10.1007/s00396-024-05333-z)
Supplement: Supplementary file 1 — Supplementary file1 (DOCX 803 KB) [file 396_2024_5333_MOESM1_ESM.docx]

**Supporting Information for**

A strategy of consistent X-ray and neutron double-difference pair distribution function analysis of nanoparticle dispersions

Sabrina L. J. Thomä^1,2^, Joerg Neuefeind^3^ and Tristan G.A. Youngs^4^ and Mirijam Zobel^1,5^

^1^ Institute of Crystallography, RWTH Aachen University, Jägerstr. 17-19, 52066 Aachen, Germany

^2^ Center for X-ray Analytics, Empa – Swiss Federal Laboratories for Materials, Science and Technology, Überlandstrasse 129, CH-8600 Dübendorf, Switzerland

^3^ Neutron Scattering Science Directorate, Oak Ridge National Laboratory, 1 Bethel Valley Road, Oak Ridge, TN 37831-6475, USA

^4^ ISIS Facility, STFC Rutherford Appleton Laboratory, Harwell Oxford, Didcot, Oxfordshire, UK

^5^ JCNS-3: Neutron Analytics for Energy Research, Forschungszentrum Jülich GmbH, Wilhelm-Johnen-Straße, 52428 Jülich, Germany

Email: sabrina.thomae@empa.ch; zobel@ifk.rwth-aachen.de

[1. Details on data processing 3](#_Toc170989747)

[1.1. NIMROD 3](#_Toc170989748)

[1.2. NOMAD 3](#_Toc170989749)

[2. Synthesis of IONPs and Sample Preparation 4](#_Toc170989750)

[2.1. Synthesis of IONPs measured at NIMROD 4](#_Toc170989751)

[2.2. Synthesis of IONPs measured at NOMAD and equilibration of powder samples 5](#_Toc170989752)

[2.3. Concentrations of measured IONP-dispersions 6](#_Toc170989753)

[3. Elaboration about derivation of the pair distribution function and conversions between different representations 6](#_Toc170989754)

[4. Total composition and *D(r)* data of IONP powders investigated at NIMROD 10](#_Toc170989755)

[5. Supplementary Information and Data for Experiment at NOMAD 13](#_Toc170989756)

[5.1. Complementary X-ray data 13](#_Toc170989757)

[5.2. Experimental details on fitting of neutron PDF of NOMAD powder data 15](#_Toc170989758)

[5.3. Fit values for atomic neutron PDF fit of NOMAD powder data 15](#_Toc170989759)

[5.4. Detailed Information on mPDF fits 17](#_Toc170989760)

[References 20](#_Toc170989761)

# Details on data processing

## NIMROD

Data was processed with GudrunN, which is the software package for neutron diffraction data reduction developed and used at ISIS. All corrections, such as correction for empty instrument, empty cell, multiple scattering, recoil effects, absorption etc., as well as normalization to a vanadium niobium standard to obtain intensities on an absolute scale, were performed within the GudrunN software. The data was processed for a *Q*-range of 0.6 –50 Å^-1^ and the differential interference cross section *F(Q)* in *barns sr^-1^ atom^-1^* as well as the differential correlation function *D(r)* in *barns atom Å^-2^* were evaluated.

## NOMAD

The data was processed with the instrument software ADDIE^1^ for a *Q*-range of 0.5 – 50 Å^-1^. With the ADDIE software the correction for the significant incoherent scattering background for hydrogen containing samples is conducted by applying a non-linear least squared fit to a pseudo-Voigt function. Further, the scattering from the empty sample container is subtracted. In this first step, *A[S(Q)-1]* and *A[g(r)-1]* normalized to the intensity at high *Q* in reciprocal space are obtained. This data is then normalized by applying a Fourier Filter to the *S(Q)-1* data to determine the factor *A*. The Fourier filter fits a scale factor to make the pair distribution function *g(r)* closest to zero on average for a specified *r*-range (e.g. below 0.85 Å), and back Fourier transforms it to obtain a normalized *S(Q)-1*. Thereby, different *Q_max_* values for different *r*-ranges were chosen, since sharp features in real space are extended features in reciprocal space and vice versa. The *Q_max_* values used for the respective *r*-ranges for the Fourier Filters are listed in Table S1. Below the first *r*-value *g*(*r*) is fitted to be 0. The Fourier filters were applied using the instrument-specific interactive data language (IDL) codes as frequently applied in studies^2, 3^ conducted at NOMAD.

Table S1: Q_max_ values for the different r-ranges in the applied Fourier Filters.

| **sample** | ***r*-range**  **Å** | ***Q_max_***  **Å^-1^** |
| --- | --- | --- |
| D_2_O | 0.85-1.35, 1.35-3.2, 3.2-157 | 49.4, 25, 13 |
| H_2_O:D_2_O | 0.85-1.35, 1.35-3.2, 3.2-157 | 49.4, 25, 13 |
| IONP-cit in D_2_O | 0.85-1.35, 1.35-3.2, 3.2-157 | 49.4, 25, 13 |
| IONP-cit in H_2_O:D_2_O | 0.85-1.35, 1.35-3.2, 3.2-157 | 49.4, 25, 13 |
| dry powder | 0.85-4; 4-11; 11-25; 25-157; | 31, 25, 17, 12 |
| D_2_O wet powder | 0.85-4; 4-11; 11-25; 25-157; | 31, 25, 17, 12 |
| H_2_O wet powder | 0.85-4; 4-11; 11-25; 25-157; | 31, 25, 17, 12 |

# Synthesis of IONPs and Sample Preparation

## Synthesis of IONPs measured at NIMROD

Two different batches of IONP powders were prepared according to our standard procedure^4^ adapted from Caruntu and Qu *et al.^5, 6^* . One batch was capped with sodium citrate (cit) and one was capped with phosphocholine (phos). The samples are therefore referred to as IONP-cit and IONP-phos.

For both batches, a solution of 32 mmol NaOH (40 g/mol) in 80 g DEG was added to a precursor solution, containing 4 mmol of FeCl_2_$\cdot$4 H_2_O (198.81 g/mol) and 8 mmol of FeCl_3_$\cdot$6 H_2_O (270.30 g/mol) in 160 g DEG, under stirring. The reaction solution was degassed under argon for two hours and subsequently heated to 220°C under reflux with a heating ramp of 130°C/h. After keeping the temperature constant for two hours the reaction solution was cooled down. At ca 100°C, either 5 mmol of sodium citrate dissolved in circa 2 mL water or 4 mmol of phospholine choride calcium salt tetrahydrate dispersed in circa 5 mL DEG was added and further stirred until the solution reached room temperature.

The IONP powder was precipitated by the addition of circa double the volume of acetone and collected on a magnet. After removal of this supernatant, the IONP powder was washed with ethanol absolute for five times in case of IONPs-cit and two times in a mixture of ethanol and ethyl acetate in case of IONPs-phos. Both powders were re-dispersed in D_2_O after the last purification step and freeze-dried. The freeze-dried powder were sent to ISIS and measured as powders as prepared and re-dispersed with a concentration of circa 100 mg/mL onsite for measurements of the dispersions.

## Synthesis of IONPs measured at NOMAD and equilibration of powder samples

Six batches of IONP powder were prepared as described in 2.1. for IONP-cit. After purification this powder was left to dry in air in the fume hood. All batches were then characterised with DLS and since a comparable diameter was obtained for all of them the powders were unified. The IONP powder was dissolved in water at a concentration of 45 mg/mL and dialysed against water for ca. 19 hours, and another 6 hours after water-change in order to remove spare citrate, residual DEG and chloride from synthesis. After dialysis the IONP dispersion was freeze-dried in order to obtain the nanocrystalline powder.

In order to create “wet” powders with adsorbed (heavy) water and nominally “dry” ones, the powders were packed in the quartz capillaries provided by the instrument staff (only about 60 – 70 mg) and stored over open dishes with binary saturated salt solutions (H_2_O/D_2_O) in a limited air volume in a desiccator equipped with a hygrometer for two weeks. A given saturated salt solution creates a specified relative humidity.^7^

A D_2_O-wet-powder, with 3.54 g D_2_O per g IONP powder, was created over a K_2_SO_4_ heavy water solution at a relative humidity of 96%. The H_2_O-wet-powder, with 2.12 g H_2_O per g IONP powder, was created over a KNO_3_ water solution at a relative humidity of 96%. The amount of adsorbed (heavy) water was determined by the weight change after the two weeks. The dry powder was equilibrated over a LiCl water solution at 11% RH. This powder lost weight upon drying in the desiccator, but the weight loss could not be tracked exactly, because the glass capillary was damaged a bit upon closing. The capillaries were, likewise as for the liquid samples, closed quickly with a rubber plug and epoxy resin, when they were taken out of the desiccators and then sent to Oakridge National Laboratory. According to the instrument staff all capillaries arrived without damage, but the samples were not checked for changes in weight.

2.2 mL of IONP dispersion in D_2_O and in H_2_O:D_2_O (50:50) mixture with a concentration of circa 100 mg/mL each were prepared, filled in 3 mm quartz glass capillaries, which were closed with a rubber plug and epoxy resin, and sent to the Oakridge National laboratory.

## Concentrations of measured IONP-dispersions

Table S2: Concentrations of measured IONP dispersions.

| **sample** | **concentration**  **mg/mL** |
| --- | --- |
| IONP-cit D_2_O NOMAD | 100.0 |
| IONP-cit D_2_O NIMROD | 103.9 |
| IONP-cit H_2_O-D_2_O NIMROD | 99.1 |
| IONP-cit H_2_O NIMROD | missing information |
| IONP-phos D_2_O NIMROD | 100.4 |
| IONP-phos H_2_O-D_2_O NIMROD | 99.1 |
| IONP-phos H_2_O NIMROD | 91.1 |

# Elaboration about derivation of the pair distribution function and conversions between different representations

The measured quantity in a neutron total scattering experiment is the number of neutrons scattered by the sample in an element of reciprocal space relative to the number of neutrons scattered by a completely disordered sample. The completely disordered sample is very well approximated by the different spin states of vanadium. Those have scattering length of almost equal magnitude, but opposing signs, and are fully uncorrelated. In general the number of neutrons measured has to be corrected for systematic distortions such as background scattering, neutrons absorbed in the sample or the vanadium, multiple (inside the sample) or secondary (sample to surrounding) scattering. The ratio of scattering intensities is then proportional to the differential cross section $\left( \frac{d\sigma}{d\omega} \right)$ of the sample. This quantity has the dimension of an area per solid angle, often quoted in barns per steradian (sr). It can be subdivided in a self-scattering and an interference part, the latter often called *F(Q)*. With $\sigma_{V}$ the scattering cross section of vanadium, $N_{V}$ the number of neutrons scattered from vanadium, $N_{s}$ the number of neutrons scattered from the sample, $I_{s}^{'}$ and $I_{V}^{'}$ the respective intensities, $c_{ij}$ the stochiometric coefficients of atoms *i* and *j*, $b_{i}$ and $b_{j}$ the coherent neutron scattering lengths of atoms *i* and *j*, and $s_{ij}$ the partial structure factors, we write the differential scattering cross section as a sum of partial structure factors:

| $\left( \frac{d\sigma}{d\omega} \right)=\frac{\sigma_{V}N_{V}}{{4\pi N}_{s}}\frac{I_{s}^{'}}{I_{V}^{'}}=\sum_{ij} c_{ij}b_{i}b_{j}\left( s_{ij}-1 \right)$ | ***1*** |
| --- | --- |

Above mentioned corrections to retrieve the differential cross section or the interference part are conducted within specialized data reduction software each used at different facilities and are performed either analytically in an absolute manner^8^ or via *ad hoc* methods^9-11^. More information on the corrections can for example be found in ^8, 12, 13^.

In the following, we will use stoichiometric factors normalized to 1 and the density as an atomic density. It is useful to define a dimensionless structure function *S(Q)* as:

| ${S(Q)}_{N}=\frac{\left( \frac{d\sigma}{d\omega} \right)}{\sum_{i} \left( c_{i}b_{i}^{2} \right)}$ | ***2*** |
| --- | --- |

The pair distribution function *g(r)* of a monoatomic system is the average number of atoms $\left\langle N\left( r \right) \right\rangle$ within an element of real space $d$*r* from a central atom relative to the number of atoms that would have been found in a completely disordered system. The symmetry of that definition with the differential cross section in reciprocal space is noted. Thus we have:

| ${g(r)}^{1 at}= \frac{<N(r)>}{4\pi\rho r^{2}dr}$ | ***3*** |
| --- | --- |

with $\rho$ is the atomic number density of the material. This *g*(*r*) has limiting values of 0 for *r=0* and 1 as *r* becomes large relative to the correlation length of the material and thus as the number of atoms becomes equal to the totally disordered system. In a multi-atom system only a weighted sum of partial pair distribution functions can be determined by diffraction methods, with each of the partials $g_{ij}$ defined similarly to equation ***3*** with the same limiting values of 0 and 1:

| $g(r)=\sum w_{ij}g_{ij}$ | ***4*** |
| --- | --- |

If the weights $w_{ij} are normalized as \sum w_{ij}=1,$ the (neutron) weighted pair distribution function also has the limits of 0 and 1 at short and long distances. For normalized weights we have:

| $w_{ij}=\frac{\left( 2-\delta_{ij} \right)c_{i}c_{j}b_{i}b_{j}}{\left( \sum c_{i}b_{i} \right)^{2}}$ | ***5*** |
| --- | --- |

$\delta_{ij}$ is the Kronecker delta, and the factor (2-$\delta_{ij}$) ensures double counting of the cross terms and single counting of the diagonal elements. *g(r)* is related by sine Fourier transform to *S(Q)* by:

| $\boldsymbol{r[g}\left( \boldsymbol{r} \right)\boldsymbol{-1]=}\frac{\sum\left( \boldsymbol{c}_{\boldsymbol{i}}\boldsymbol{b}_{\boldsymbol{i}}^{\boldsymbol{2}} \right)}{\boldsymbol{2}\boldsymbol{\pi}^{\boldsymbol{2}}\boldsymbol{\rho}\left( \sum\boldsymbol{c}_{\boldsymbol{i}}\boldsymbol{b}_{\boldsymbol{i}} \right)^{\boldsymbol{2}}}\int\boldsymbol{Q}\left[ \boldsymbol{S}\left( \boldsymbol{Q} \right)\boldsymbol{-1} \right]\sin\boldsymbol{(Qr)}\boldsymbol{dQ}$ | ***6*** |
| --- | --- |

The left hand side of equation ***6*** is beneficial for the investigation of nanostructured materials since it emphasizes structural features at longer distances and is often (somewhat confusingly) simply called *PDF(r)*:

| $PDF\left( r \right)=r\cdot\left[ g(r)-1 \right]$ | ***7*** |
| --- | --- |

For the data acquired at NOMAD at SNS this total scattering structure function *S(Q)* as well as the PDF in the form *PDF(r)* were processed with the beamline software ADDIE^1^ and are presented and discussed within this article.

NIMROD data were processed with GudrunN^8^ and are presented as the interference differential cross section *F(Q)*, in units of *barns sr^-1^ atom^-1^* and the differential correlation function *D(r)* in units of *barns atom Å^-2^*. For these functions the weight factors, *c*_i_ are not normalized and they differ by constant factors from *S(Q)-1* and *g(r)* and respectively^14^. Their absolute magnitudes also depend on the units used:

| $F\left( Q \right)= \sum{(c_{i}b_{i})}^{2}\cdot\left[ S\left( Q \right)-1 \right]$ | ***8*** |
| --- | --- |
| $D(r)= 4\pi r\rho\sum{(c_{i}b_{i})}^{2}\cdot\left[ g(r)-1 \right]$ | ***9*** |

A commonly used form of the PDF by a community investigating crystalline disordered materials is also the ‘reduced’ pair distribution function *G(r)^12^*, which relates to *PDF(r)* by:

| $G(r)= 4\pi\rho PDF(r)$ | ***10*** |
| --- | --- |

This is also the form, which is expected by the modelling software *PDFgui^15^*, which was also used in this article. However, in *PDFgui^15^* a scale factor is refined freely for the data set and thus the respective use of *PDF(r)*, *D(r)* and *G(r)* is less critical^14^. It should also be noted that the atomic density $\rho$ for our IONPs has a considerable level of uncertainty due to the significant amount of organics, and that this uncertainly is captured by the scale factor.

Since neutrons scatter from the nucleus, in a typical total scattering experiment using unpolarized neutrons the measured scattered intensity is the sum of nuclear and magnetic scattering.^16, 17^ Therefore, in analogy to the atomic PDF, also a magnetic PDF (mPDF) can be gained, where peaks represent spin-pair correlations of ferromagnetically coupled spins (positive) and antiferromagnetically coupled spins (negative). Consequently, this mPDF *f(r)* can be obtained by a sine Fourier transform from the magnetic scattering intensity: ^17, 18^

| $f\left( r \right)= \frac{2}{\pi}\int_{0}^{\infty} Q\left( \frac{I_{\mathrm{mag}}}{\frac{2}{3}N_{s}S\left( S+1 \right)\left( \gamma r_{0} \right)^{2f_{m}^{2}\left( Q \right)}}-1 \right)sin(Qr)dQ$ | ***11*** |
| --- | --- |
| $f\left( r \right)= \frac{1}{N_{s}}\frac{3}{2S(S+1)}\sum_{i \neq j} ( \frac{A_{ij}}{r}\delta\left( r-r_{ij} \right)+B_{ij}\frac{r}{r_{ij}^{3}}\Theta\left( r_{ij}-r \right))$ | ***12*** |

Here, *I*_mag_ is the orientationally averaged magnetic scattering intensity, *S* is the spin quantum number, the subscripts *i* and *j* refer to individual magnetic moments *S_i_* and *S_j_* separated by a distance $r_{ij}$, *r*_o_ is the classical electron radius, $\gamma$ is the neutron magnetic moment in units of nuclear magnetons, *f*_m_*(Q)* is the magnetic form factor, *N_s_* is the number of spins in the system, $\delta$ denotes a delta function, *Θ* is the Heaviside step function and *Q* is the magnitude of the scattering vector. The coefficients *A_ij_* = $\left\langle S_{i}^{y}S_{j}^{y} \right\rangle$ and *B_ij_* = *2* $\left\langle S_{i}^{x}S_{j}^{x} \right\rangle-\left\langle S_{i}^{y}S_{j}^{y} \right\rangle$ give information about the alignment of the magnetic moments. To express them a coordinate system is defined locally for each spin pair such that $\hat{x}$ lies along the vector joining the pair of spins and $\hat{y}$ is chosen such that the *xy*-plane contains the pair axis and the first spin.

To obtain this mPDF $f(r)$, the magnetic scattering needs to be isolated in reciprocal space, normalized by the magnetic form factor and Fourier transformed. Yet, neutron total scattering data can also be reduced according to standard atomic PDF protocols, which results in $G_{tot}\left( r \right)(\mathrm{or}{PDF}_{\mathrm{tot}}\left( r \right), D_{tot}\left( r \right))$, an additive sum of the atomic PDF and an unnormalized mPDF $d(r)$ due to lacking division by the magnetic form factor. It can be described as:^17^

| $G_{tot}\left( r \right)=Ϝ \left\{ Q\left( \frac{I_{\mathrm{nuc}}}{N_{a}\left\langle b \right\rangle^{2}}-\frac{\left\langle b^{2} \right\rangle}{\left\langle b \right\rangle^{2}} \right) \right\}+Ϝ\left\{ Q\frac{I_{\mathrm{mag}}}{N_{a}\left\langle b \right\rangle^{2}} \right\}$ | ***13*** |
| --- | --- |
| $G_{tot}\left( r \right)= = G\left( r \right)+ \frac{d(r)}{N_{a}\left\langle b \right\rangle^{2}}$ | ***14*** |

$Ϝ\left\{ \ldots\right\}$is a shorthand for Fourier transform and includes a constant prefactor, *I*_nuc_ is the nuclear scattering intensity, *N*_a_ is the number of atoms and *b* is again the coherent (nuclear) scattering length. The lacking normalization to the magnetic form factor gives rise to an additional peak at low *r* in the unnormalized mPDF $d\left( r \right)$, which does not arise due to pairwise magnetic correlation, and broadens *d*(*r*) by twice the spin density.^17^ Yet, $d\left( r \right)$ can be easily obtained as residual of an atomic neutron PDF refinement of a magnetic material and can then be refined subsequently^17, 19, 20^.

# Total composition and *D(r)* data of IONP powders investigated at NIMROD

In order to determine the total composition of the IONP powders some additional characterization (elemental analysis, thermogravimetric analysis, atomic absorption spectroscopy and density measurement) was conducted.

Elemental analysis was performed on 1.7 – 3.0 mg of the nanocrystalline powder with an Elementar vario EL III (Elementar, Germany) providing the C/H/N contents of the samples.

Thermogravimetric analysis (TGA) measurements were performed from 30 – 1000 °C on a STA PT16000 (Linseis, Germany) with a heating ramp of 10 °C min^-1^ under argon atmosphere.

The iron content of the samples was determined using flame atomic absorption spectroscopy (AAS) with a Varian AA100 (Varian, USA). For the measurements 2.0 – 2.5 mg of powder were dissolved in 5 mL concentrated HCL and diluted with Milli-Q water to 100 mL.

The density of the powders was determined in the 10 cm^3^ chamber of an AccuPyc 1330 gas pycnometer (Micromeritis, USA) operated with helium gas.

The total composition of the sample was determined by measuring the Fe content with AAS and the C, H and N content with elemental analysis. For the phos capped powder the P and O content from organics was then determined from the measured N content with the known chemical composition of phos (C_5_H_15_NO_4_P). The measured content and resulting total composition of IONP-phos are given in Table S3.

Table S3: The amount of the elements contained in IONP-phos powder are listed in wt% together with the method they were determined with and the resulting total composition at the bottom of the table.

The total amount of organic content from CHN analysis is compared to the organic content determined with TGA. For the elements C and H the expected amounts with respect to the N content and the assumed composition of the ligand of C_5_H_15_O_4_P is given.

| **element** | **content wt%** | **expected wt%** | **method** |
| --- | --- | --- | --- |
| Fe | 47.2 |  | AAS |
| C | 4.72 | 4.87 | CHN |
| H | 1.43 | 1.23 | CHN |
| N | 1.14 |  | CHN |
| P | 2.52 |  | CHN |
| O (total) | 42.6 |  | rest |
| O (ligand) | 5.21 |  | CHN |
| sum organics | 15.02 |  | CHN |
| organics | 15.00 |  | TGA |
| **total composition: Fe_1.00_C_0.46_H_1.66_N_0.1_P_0.1_O_3.12_** | | | |

In addition, C and H contents, as they would be expected from measured N content, and organic content of the sample determined with TGA analysis are given for comparison. According to the measured N content, the C and H content were slightly lower than expected, but in absolute value this deviation was in µg-range ($<$ 5 µg) and thus in the range of the error of the method. Since the H content was not higher as expected from C and N content, it can be assumed that this nominally “dry” sample does not contain a significant amount of surface water. Organic content determined from CHN and TGA analysis are in perfect agreement.

In Table S4 the total composition of IONP-cit is listed. Since no heteroatom is present in the ligand, in this case the H content can only be calculated with respect to the measured C content according to the composition of citrate (C_6_H_5_O_7_). The deviation between measured and expected H content suggests, that besides surface water also residual DEG from synthesis could be present.

Table S4: The amount of the elements contained in IONP-cit is listed in wt%.

The total amount of organic content from CHN analysis is compared to the organic content determined with TGA. For the element H the expected amounts with respect to the C content and the assumed composition of the ligand of C_6_H_5_O_7_ is given.

| **element** | **content wt%** | **expected wt%** | **method** |
| --- | --- | --- | --- |
| Fe | 66.22 |  | AAS |
| C | 3.79 |  | CHN |
| H | 1.02 | 0.27 | CHN |
| O (total) | 28.97 |  | rest |
| O (ligand) | 11.87 |  | CHN |
| sum organics | 16.87 |  | CHN |
| organics | 15.00 |  | TGA |
| **total composition: Fe_1.00_C_0.27_H_0.85_O_1.53_** | | | |

Since at NIMROD the data is processed on an absolute scale as the total interference cross section (*F*(*Q*)) in barns sr^-1^ atom^-1^ by normalization to a vanadium niobium standard and then Fourier transformed to the differential correlation function *D*(*r*) the presented determination of the exact sample composition of IONP powders is crucial. Figure S1a) exemplarily highlights the impact of neglecting organics compared to data processing with the total composition and measured mass density of the sample for the case of IONP-phos powder. Only after assuming the total composition a reasonable progression of the -4$\pi r\rho$ baseline and intensity ratio of the correlation peaks was obtained. All data sets contain a termination ripple at 0.85 Å, which was eliminated later in the processing. In Figure S1c) and d) complementary X-ray data taken at our lab diffractometer^21^ and processed with PDFgetX3^22^ is shown. In this case assuming either a composition of Fe_3_O_4_ or the retrieved total composition does not influence the data strongly – see Figure S1c). The X-ray PDFs obtained from PDFgetX3^22^ are arbitrarily scaled and possess a constant scale set-off due to the ad-hoc approach used for the data correction^23^, but the scaling of the peaks to one another is preserved. That is the reason why the data from processing with different assumed composition can be almost perfectly scaled to each other by multiplication with a scale factor (0.6 - cf. Figure S1c). Figure S1b) shows measured *D(r)* at NIMROD of IONP powders in comparison. It is evident that the dampening related to instrumental resolution is strong, since the *D*(*r*) are declined to zero already at about 40 Å, even though the X-ray PDF is declined to zero only at about 70 Å, see Figure S1d). Due to this severe dampening and lacking instrumental resolution (liquid dedicated instrument) at NIMROD, this powder data was solely used for subtraction in the double-difference treatment and not further analysed (modelled).


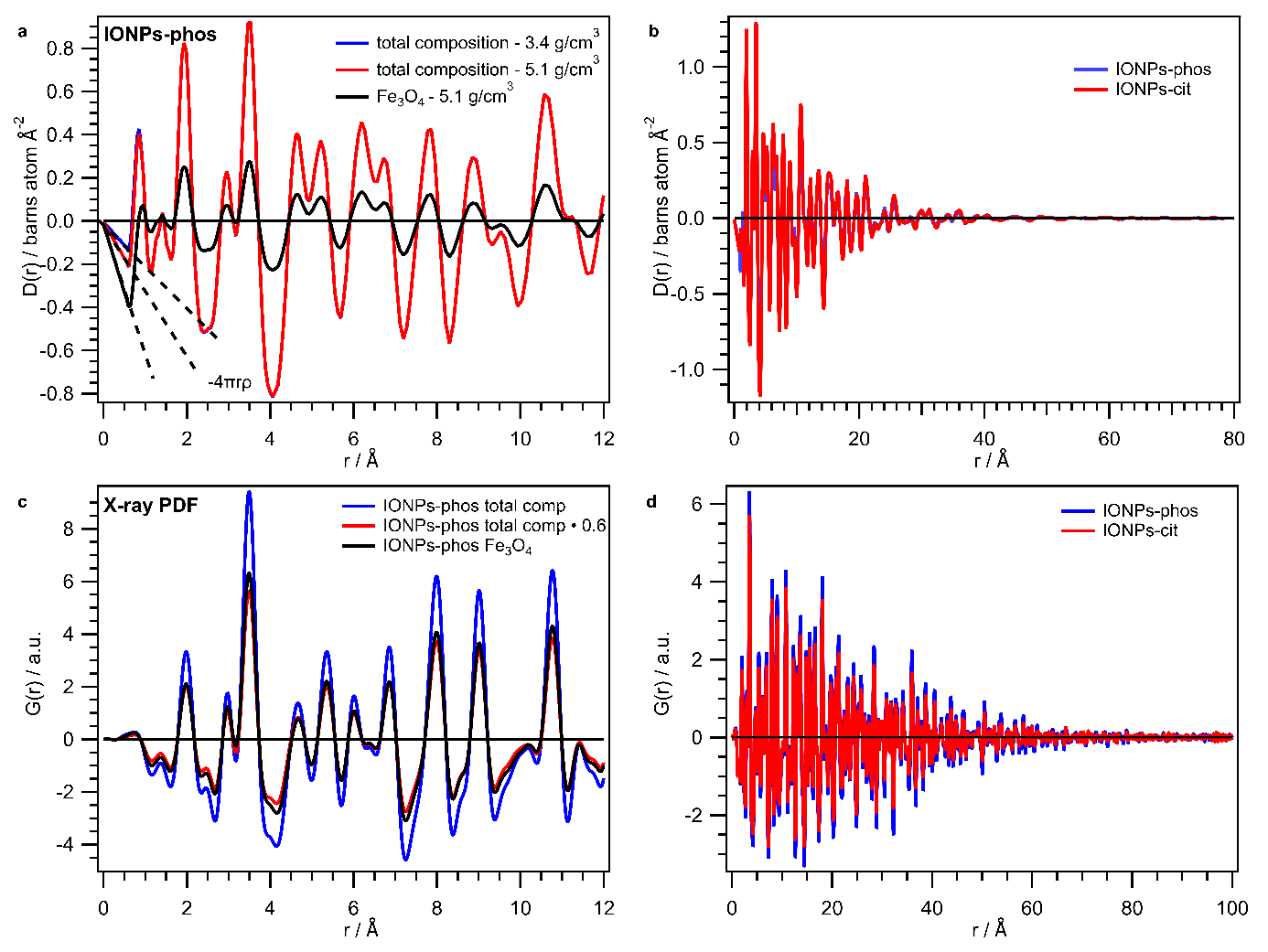


Figure S1: Influence of assumed composition for D(r) of IONP powders investigated at NIMROD in comparison to for X-ray PDF data.

a) Influence of the considered composition and mass density of IONP-phos powder during data processing on D(r). b) D(r) of IONP powders investigated at NIMROD for different ligands c) Influence of considered composition of IONP-phos data for X-ray PDF processed with PDFgetx3. d) X-ray PDF of IONPs powders investigated at NIMROD for different ligands.

# Supplementary Information and Data for Experiment at NOMAD

## Complementary X-ray data

X-ray PDF data of the nanocrystalline powder in a 1 mm diameter Kapton capillary was taken at ID31 of the European Synchrotron Radiation Facility (ESRF) in Grenoble. The sample was measured in rapid acquisition PDF mode with the Pilatus X2M detector (Dectris) at an energy of 65 keV. For distance calibration and determination of instrumental resolution cerium oxide standard was used (*Q_damp_*= 0.0128 Å^-^1; *Q_broad_*= 0.0132 Å^-1^). The PDF was processed with PDFgetX3^22^ and modelled with the same maghemite phase^24^ with PDFgui^15^.

The X-ray data is shown in Figure S2:


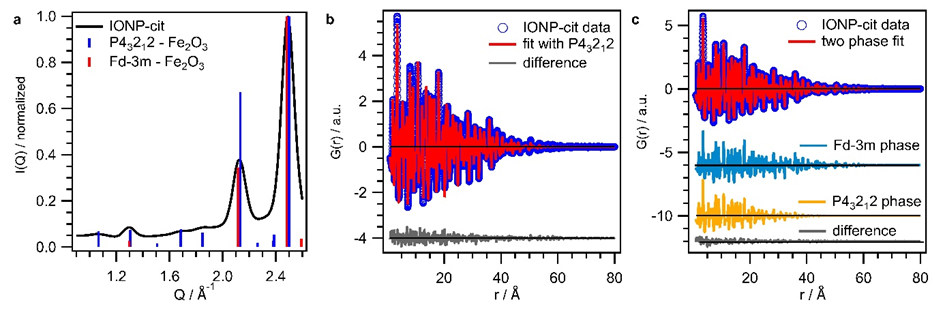


Figure S2: Complementary X-ray data for IONP powder investigated at NOMAD.

a) I(Q) data of IONP-cit powder (NOMAD) indexed with maghemite structure in P4_3_2_1_2 phase and Fd$\bar{3}$m phase. b) X-ray PDF of IONP-cit powder (NOMAD) modelled with P4_3_2_1_2 phase. c) X-ray PDF of IONP-cit powder (NOMAD) modelled with a two-phase fit (P4_3_2_1_2 and Fd$\bar{3}$m).

Figure S2a) displays the *I(Q) vs Q* pattern of the IONP-cit powder (NOMAD) indexed with two different models for the maghemite structure. Both patterns are simulated with occupancies corresponding to a nominal composition of Fe_2_O_3_. For the P4_3_2_1_2 model this is a occupancy of 0.33 on Fe(4) position and for the Fd$\bar{3}$m model the occupancy on the tetrahedral Fe site is 0.83. The additional reflexes arising due to vacancy ordering in the tetragonal model are slightly visible. Yet, with the occupancy of 0.33 on the Fe(4) site the intensity ratios of the reflexes in the *Q*-range up to 2.1 Å^-1^ do not match the data. Consequently, the IONPs either possess a non-stochiometric composition in between Fe_2_O_3_ and Fe_3_O_4_ or are core-shell particles with a Fe_3_O_4_ core and an oxidized shell. The X-ray PDF retrieved from the total scattering data was modelled with P4_3_2_1_2 phase (see Figure S2b)) as well as a two-phase model with P4_3_2_1_2 and Fd$\bar{3}$m phase (Figure S2c)). Refined values are the unit cell parameters (a,c), the scale factor to match theoretical and experimental PDF data in intensity (scale), the isotropic displacement parameters (u_iso_ for Fe and O), a parameter to account for correlated atomic motion (delta 2), the spherical diameter of the particles to account for the dampening of the PDF, as well as the occupancies of the Fe and O sites. The values are listed in Table S6. With the two-phase fit the goodness of fit value (R_w_) could be significantly improved. The obtained fit values are best rationalized with IONPs possessing a non-stochiometric composition with a structurally coherent transition from Fe-rich core and a more oxidized outer region. The overall Fe/O ratios for the two fits are 0.68 (P4_3_2_1_2) and 0.72 (two-phase).

Table S5: Fit values for X-Ray PDF fit of IONP-cit powder (NOMAD) with P4_3_2_1_2 model and a two-phase fit of P4_3_2_1_2 and Fd$\bar{3}$m in comparison. In case the cell is empty the parameter either does not apply for the model or was not refined.

| **parameter** | **fit with P4_3_2_1_2** | **two-phase fit** | |
| --- | --- | --- | --- |
|  |  | **Fd**$\bar{\boldsymbol{3}}$**m** | **P4_3_2_1_2** |
| **a** (Å) | 8.3724 | 8.3542 | 8.3956 |
| **c** (Å) | 8.3210 |  | 8.3008 |
| **scale** | 0.6289 | 0.3041 | 0.3514 |
| **delta 2** (Å^2^) | 2.5820 | 3.1469 | 2.6192 |
| **u_iso_ Fe** (Å^2^) | 0.0053 | 0.0092 | 0.0053 |
| **u_iso_ O** (Å^2^) | 0.0157 | 0.0109 | 0.0267 |
| **spherical diameter** (Å) | 61.0584 | 74.0489 | 50.1961 |
| **cccupation Fe(4) / tetrahedral** | 0.4576 |  | 0.3595 |
| **occupation Fe octahedral** |  | 0.7714 |  |
| **occupation O** |  | 0.8140 |  |
| **O_z_ position** (Å) |  | -0.6304 |  |
| **phase content atoms** |  | 0.4410 | 0.5590 |
| **R_w_** (goodness of fit) | 0.136 | 0.091 | |

## Neutron PDF of NOMAD powder data with experimental details on modelling


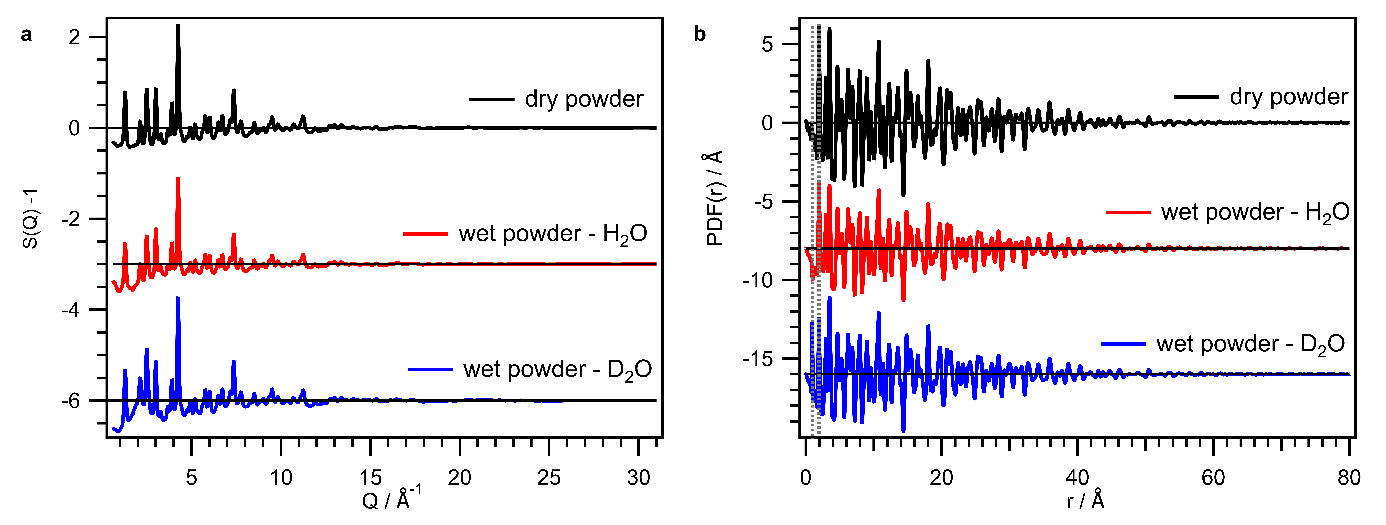


Figure S3: a) S(Q) – 1 data of the three powders investigated at NOMAD. b) Respective PDF(r) data of the three investigated powders at NOMAD.

In Figure S3 the data of the three powder samples investigated at NOMAD are shown. In panel a) the *S(Q) -1* data are shown and distinctive differences in the intensity ratios of the reflexes between 1.2 – 2.5 Å^-1^ (also discussed for the X-ray data) are detected. In panel b) the respective *PDF(r)* data is shown, revealing a OD/OH distance correlation for the wet powders, as well as varied intensity amongst the three powders of the first Fe-O distance correlation at 1.94 Å (dashed grey lines).

The atomic PDFs of the powder samples were modelled with a maghemite phase described in tetragonal symmetry^24^ and an additional “dummy pair” OD/OH phase in case of wet powders according to Wang *et al*.^25^ and also applied by Plekhanov *et al.^26^* using the PDFgui^15^ software package. Instrumental resolution parameters *Q_damp_*= 0.018 Å^-1^ and *Q_broad_*= 0.019 Å^-1^ were provided by the instrument staff. The observed difference between experimental data and fit was then modelled with the diffpy.mpdf package, which is an extension of the Diffpy-CMI library^27^.

## Fit values for atomic neutron PDF fit of NOMAD powder data

The fit values for the atomic neutron PDF fits of the three investigated IONP-cit powders with P4_3_2_1_2 phase are listed in Table S7. A two-phase fit as conducted for the X-ray PDF data did not improve the goodness of fit value for any of the three cases.

The scale factor is used to match *PDF(r)* with the *G(r)* expected by *PDFgui.* This scale factor is close to 1 for the dry powder, since $4\pi\rho$ for the modelled iron oxide phase (Fe_0.4_O_0.6_ with molecular mass of 32 g mol^-1^ and density of 4.9 g cm^-3^) is 1.156 atoms Å^-3^. The model does not account for the contained organics, which reduces $\rho$ of our investigated sample and accordingly the constant factor $4\pi\rho$.

Table S6: Fit values for the atomic neutron PDF fits of three analysed IONP-cit powders in comparison.

| **parameter** | **dry** | **D_2_O wet** | **H_2_O wet** |
| --- | --- | --- | --- |
| **a** (Å) | 8.3779 | 8.3777 | 8.3784 |
| **c** (Å) | 8.3116 | 8.3149 | 8.3147 |
| **scale** | 1.0368 | 0.8130 | 0.7397 |
| **delta 2** (Å^2^) | 2.9131 | 2.8132 | 2.8402 |
| **u_iso_ Fe** (Å^2^) | 0.0058 | 0.0064 | 0.0062 |
| **u_iso_ O** (Å^2^) | 0.0073 | 0.0080 | 0.0076 |
| **spherical diameter** (Å) | 60.4459 | 60.8866 | 61.7929 |
| **occupation Fe(4)** | 0.45 (fixed) | 0.45(fixed) | 0.45 (fixed) |
| **OD/OH dummy phase** |  |  |  |
| **a** (Å) |  | 3.8091 | 4.3611 |
| **scale** |  | 0.6608 | 0.0203 |
| **u_iso_ O** (Å^2^) |  | 0.0016 | 0.0134 |
| **u_iso_ D/H** (Å^2^) |  | 0.6608 | 0.0134 |
| **R_w_** (goodness of fit) | 0.190 | 0.187 | 0.201 |

In Figure S4 the fits for the atomic PDF of the nominally dry IONP-cit powder are broken into two ranges (0.5 – 25 Å and 25 – 50 Å) and compared to the fit conducted over the whole *r*-range. Corresponding fit values are listed in Table S8. With the fit broken into two *r*-ranges the resulting difference curves could not be significantly minimized and the goodness of fit parameter is not significantly improved (cf. Figure S4 and Table S8).


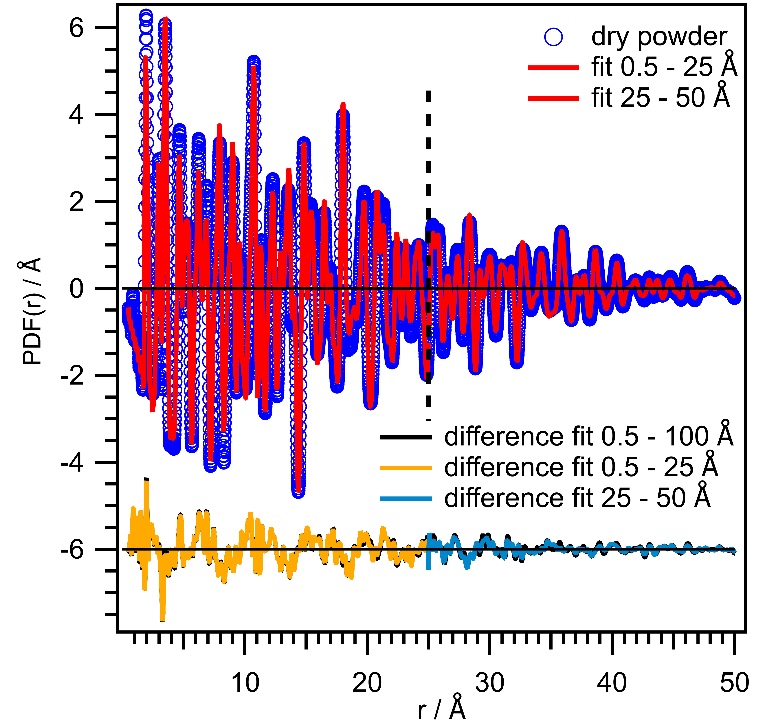


Figure S4: Atomic neutron PDF fit for the nominally dry IONP-cit powder conducted over two different ranges (0.5 – 25 Å and 25 – 50 Å).

The differences for the two fits (0.5 – 25 Å – yellow; 25 – 50 Å light blue) are shown in offset (-6) in comparison to the difference for the fit conducted over the whole r-range (black) of interest (0.5 – 100 Å).

Table S7: Fit values for the atomic neutron PDF fit for the nominally dry IONP-cit powder for three different ranges (0-100 Å; 0.5 – 25 Å and 25 – 50 Å) in comparison.

| **parameter** | **0.5 – 100 Å** | **0.5 – 25 Å** | **25 – 50 Å** |
| --- | --- | --- | --- |
| **a** (Å) | 8.3779 | 8.3760 | 8.3685 |
| **c** (Å) | 8.3116 | 8.3150 | 8.3328 |
| **scale** | 1.0368 | 1.0597 | 1.0965 |
| **delta 2** (Å^2^) | 2.9131 | 2.8562 | fixed |
| **u_iso_ Fe** (Å^2^) | 0.0058 | 0.0057 | 0.0105 |
| **u_iso_ O** (Å^2^) | 0.0073 | 0.0073 | 0.0086 |
| **spherical diameter** (Å) | 60.4459 | 56.0308 | 61.8981 |
| **occupation Fe(4)** | fixed | fixed | fixed |
| **R_w_** (goodness of fit) | 0.190 | 0.185 | 0.189 |

## Detailed Information on mPDF fits

In Figure S5 the overall fits (atomic and magnetic contribution) for the three investigated IONP-cit powders are shown.


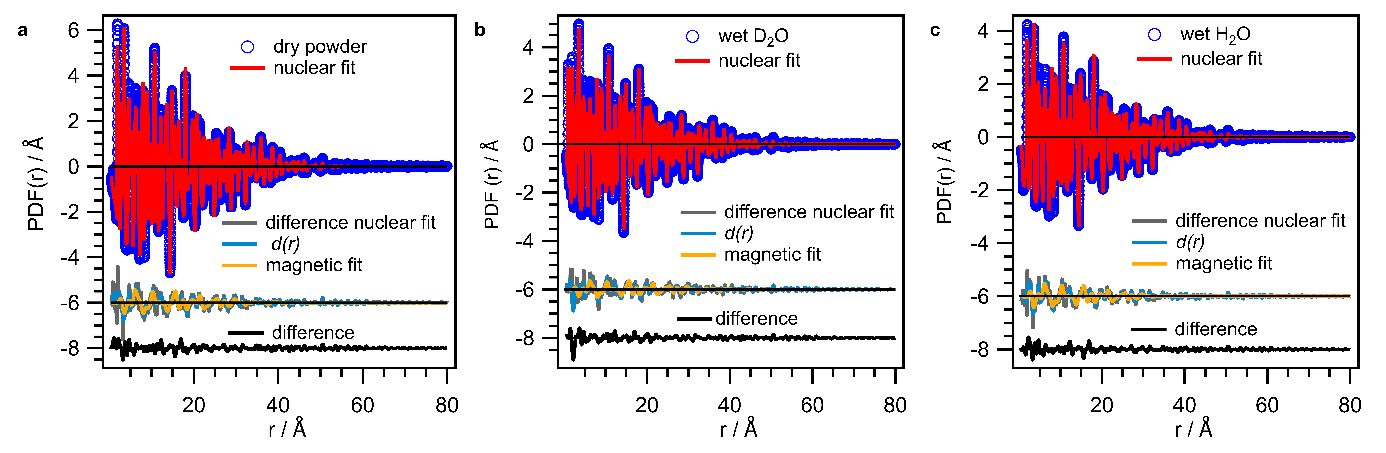


Figure S5: PDF(r) data of IONP powders (dry in a), wet D_2_O in b), wet H_2_O in c)) with their fits for nuclear and magnetic contributions over an r-range up to 80 Å.

Measured reduced PDF data is given in blue, its nuclear fit in red and the resulting difference in grey in offset at -6. The resulting difference was smoothed to obtain d(r) (light blue in offset, -8) and fitted with a magnetic model (yellow in offset, -6). The overall difference is given in black in offset -8.

After modelling the measured data with the atomic models as described above in section 5.3 the grey difference curves (offsest -6) were obtained. These signals were smoothed by convolution of the full Fourier transform with a sinc function by an approach listed in the diffpy.mPDF package and also applied for the mPDF fit in Andersen *et al*^19^. The smoothing is justified by the fact, that high frequency noise arises due to scattering at high *Q*-values, where the magnetic scattering contribution is negligible due to the *Q*-dependency of the magnetic form factor. According to equation ***14***, the smoothed signal is denoted as *d*(*r*) and represents the unnormalized mPDF. It was modelled with the ferrimagnetic structure of reference^19^, similar to bulk magnetite/maghemite. The model consists of a sublattice for the tetrahedral Fe sites and a sublattice for octahedral Fe sites with three different magnetic Fe species, since the occupancy on one of the Fe sites is lowered. Thus, the magnetic moment for both sites are refined independently. The magnetic moment is calculated by calibrating the refined value for the spin vector lengths in the model with the average scattering length, nuclear scale factor, fraction of magnetic atoms and occupancy of respective site. Further, a spherical shape function^28^ to model the magnetic domain size is included. The net magnetic moment of the material is accounted for with a term linear in *r* with negative slope.^19, 20, 29^ The spins were thereby fixed to lie in the $\left\langle111 \right\rangle$ direction like for bulk Fe_3_O_4_ and $\gamma$-Fe_2_O_3_ as also performed by Andersen*et al.^19^*. The reason for this fixing was that ordering along other crystallographic directions resulted in unreasonably high magnetic moments. The refined parameters, to summarize magnetic moments for the tetrahedral and octahedral Fe sites (µ_tet_ and *µ_oct_*), magnetic spherical domain size and slope, as well as goodness of fit values are listed in Table S8. Additionally, the resulting average magnetic moment *µ_avg_* is given in Table S8 and the magnetic domain size is compared to the nuclear one.

Table S8: Fit values for the mPDF fits with goodness of fit value.

Retrieved magnetic domain size is compared to the nuclear domain size.

| **parameter** | **dry** | **wet D_2_O** | **wet H_2_O** |
| --- | --- | --- | --- |
| **µ_avg_** (µ_B_) | 4.20 ± 0.04 | 3.85 ± 0.06 | 4.55 ± 0.05 |
| **µ_tet_** (µ_B_) | 4.38 ± 0.07 | 4.39 ± 0.07 | 4.53 ± 0.09 |
| **µ_oct_** (µ_B_) | 4.10 ± 0.02 | 3.54 ± 0.05 | 4.56 ± 0.03 |
| **slope** | 1.8$\cdot$10^-5^ ± 2.3$\cdot$10^-4^ | 2.9$\cdot$10^-4^ ± 1.9$\cdot$10^-4^ | 4.6$\cdot$10^-24^ ± 2.4$\cdot$10^-4^ |
| **magnetic domain size** (nm) | 4.9 ± 0.05 | 5.6 ± 0.08 | 4.3 ± 0.04 |
| **nuclear domain size** (nm) | 6.0 | 6.1 | 6.2 |
| **chi^2^** (goodness of fit) | 0.0087 | 0.0085 | 0.0056 |

From Table S8 it is obvious, that reasonable magnetic moments comparable with those from Andersen*et al.^19^* and in accordance with further literature^24, 30^ were obtained. Magnetic domain sizes were found to be 70-90% of the nuclear domain size (particle size) suggesting less spin disorder in the surface layer in comparison to Andersen*et al.^19^*. It should be mentioned, that the magnetic domain size and magnetic moment are correlated and counteracting. The two parameters, magnetic domain size and magnetic moment, are showing a reverse trend (see Table S8). The H_2_O wet powder exhibits the smallest magnetic domain size, but the highest magnetic moments and D_2_O wet powder the biggest magnetic domain size and smallest magnetic moment.

In Figure S6 two mPDF fits (free refinement and magnetic domain size fixed) conducted for the H_2_O wet IONP powder investigated at NOMAD are shown in comparison. With the magnetic domain size fixed to the average value, also a magnetic moment lying in between the values for the other two samples was obtained confirming the strong correlation of the two parameters. Fit values for both fits are listed in Table S9.


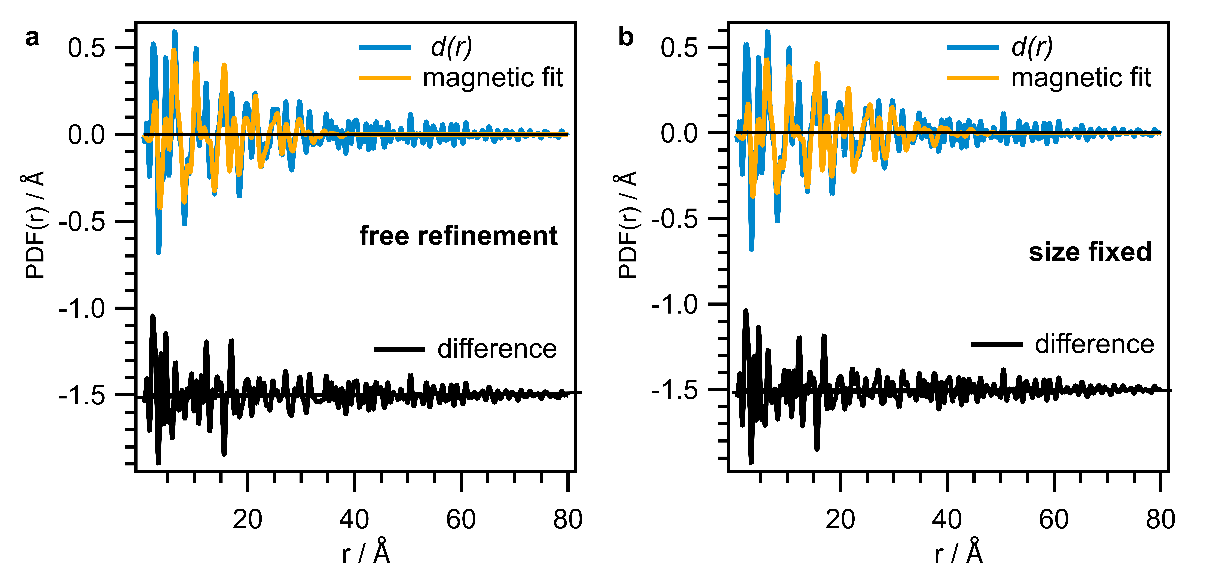


Figure S6: mPDF fit for IONP powder with adsorbed H_2_O.

In a) with free refinement in comparison to in b) with fixed magnetic domain size.

Table S9: Fit values for the mPDF fit of the sample with adsorbed H_2_O for “free refinement” and conducted with fixed magnetic domain size in comparison.

| **parameter** | **wet H_2_O**  **free refinement** | **wet H_2_O**  **magnetic domain size fixed** |
| --- | --- | --- |
| **µ_avg_** (µ_B_) | 4.55 ± 0.05 | 4.12 ± 0.05 |
| **µ_tet_** (µ_B_) | 4.53 ± 0.09 | 4.04 ± 0.08 |
| **µ_oct_** (µ_B_) | 4.56 ± 0.03 | 4.16 ± 0.03 |
| **slope** | 4.6$\cdot$10^-24^ ± 2.4$\cdot$10^-4^ | 3.7$\cdot$10^-13^ ± 1.7$\cdot$10^-4^ |
| **magnetic domain size** (nm) | 4.3 ± 0.04 | fixed 5.3 |
| **nuclear domain size** (nm) | 6.2 | 6.2 |
| **chi^2^** (goodness of fit) | 0.0056 | 0.0058 |

# References

(1) McDonnell, M. T.; Olds, D. P.; Page, K. L.; Neufeind, J. C.; Tucker, M. G.; Bilheux, J. C.; Zhou, W.; Peterson, P. F. ADDIE: ADvanced DIffraction Environment – a software environment for analyzing neutron diffraction data. *Acta Crystallographica Section A Foundations and Advances* **2017**, *73* (a1), a377-a377. DOI: 10.1107/s0108767317096325.

(2) Liu, J.; Olds, D.; Peng, R.; Yu, L.; Foo, G. S.; Qian, S.; Keum, J.; Guiton, B. S.; Wu, Z.; Page, K. Quantitative Analysis of the Morphology of {101} and {001} Faceted Anatase TiO2 Nanocrystals and Its Implication on Photocatalytic Activity. *Chemistry of Materials* **2017**, *29* (13), 5591-5604. DOI: 10.1021/acs.chemmater.7b01172.

(3) Semrouni, D.; Wang, H. W.; Clark, S. B.; Pearce, C. I.; Page, K.; Schenter, G.; Wesolowski, D. J.; Stack, A. G.; Clark, A. E. Resolving local configurational contributions to X-ray and neutron radial distribution functions within solutions of concentrated electrolytes - a case study of concentrated NaOH. *Phys Chem Chem Phys* **2019**, *21* (13), 6828-6838. DOI: 10.1039/c8cp06802j.

(4) Eckardt, M.; Thoma, S. L. J.; Dulle, M.; Horner, G.; Weber, B.; Forster, S.; Zobel, M. Long-Term Colloidally Stable Aqueous Dispersions of </=5 nm Spinel Ferrite Nanoparticles. *ChemistryOpen* **2020**, *9* (11), 1214-1220. DOI: 10.1002/open.202000313.

(5) Caruntu, D.; Caruntu, G.; Chen, Y.; O'Connor, C. J.; Goloverda, G.; Kolesnichenko, V. L. Synthesis of variable-sized nanocrystals of Fe3O4 with high surface reactivity. *Chemistry of Materials* **2004**, *16* (25), 5527-5534. DOI: 10.1021/cm0487977.

(6) Qu, H.; Caruntu, D.; Liu, H.; O'Connor, C. J. Water-dispersible iron oxide magnetic nanoparticles with versatile surface functionalities. *Langmuir* **2011**, *27* (6), 2271-2278. DOI: 10.1021/la104471r.

(7) Greenspan, L. Humidity fixed points of binary saturated aqueous solutions. *Journal of Research of the National Bureau of Standards Section A: Physics and Chemistry* **1977**, *81A* (1), 89. DOI: 10.6028/jres.081A.011.

(8) Soper, A. K. *GudrunN and GudrunX: programs forcorrecting raw neutron and X-ray diffraction data to differential scattering cross section*; RAL LibrarySTFC Rutherford Appleton Laboratory, 2011.

(9) Juhás, P.; Louwen, J. N.; van Eijck, L.; Vogt, Eelco T. C.; Billinge, Simon J. L. PDFgetN3: atomic pair distribution functions from neutron powder diffraction data using ad hoc corrections. *Journal of Applied Crystallography* **2018**, *51* (5), 1492-1497. DOI: 10.1107/s1600576718010002.

(10) Neuefeind, J.; Feygenson, M.; Carruth, J.; Hoffmann, R.; Chipley, K. K. The Nanoscale Ordered MAterials Diffractometer NOMAD at the Spallation Neutron Source SNS. *Nuclear Instruments and Methods in Physics Research Section B: Beam Interactions with Materials and Atoms* **2012**, *287*, 68-75. DOI: 10.1016/j.nimb.2012.05.037.

(11) Page, K.; White, C. E.; Estell, E. G.; Neder, R. B.; Llobet, A.; Proffen, T. Treatment of hydrogen background in bulk and nanocrystalline neutron total scattering experiments. *Journal of Applied Crystallography* **2011**, *44* (3), 532-539. DOI: 10.1107/s0021889811001609.

(12) Egami, T.; Billinge, S. J. L. *Underneath the Bragg peaks - Structural Analysis of Complex Materials*; Pergamon Materials Series, 2012.

(13) Dove, M. T.; Li, G. Review: Pair distribution functions from neutron total scattering for the study of local structure in disordered materials. *Nuclear Analysis* **2022**, *1* (4), 100037. DOI: 10.1016/j.nucana.2022.100037.

(14) Peterson, P. F.; Olds, D.; McDonnell, M. T.; Page, K. Illustrated formalisms for total scattering data: a guide for new practitioners. *J Appl Crystallogr* **2021**, *54* (Pt 1), 317-332. DOI: 10.1107/S1600576720015630 From NLM PubMed-not-MEDLINE.

(15) Farrow, C. L.; Juhas, P.; Liu, J. W.; Bryndin, D.; Bozin, E. S.; Bloch, J.; Proffen, T.; Billinge, S. J. PDFfit2 and PDFgui: computer programs for studying nanostructure in crystals. *J Phys Condens Matter* **2007**, *19* (33), 335219. DOI: 10.1088/0953-8984/19/33/335219.

(16) Frandsen, B.; Billinge, S. J. L. Investigating short-range magnetic correlations in real space with the magnetic pair distribution function (mPDF). *Neutron News* **2016**, *27* (3), 14-16. DOI: 10.1080/10448632.2016.1197588.

(17) Frandsen, B. A.; Billinge, S. J. Magnetic structure determination from the magnetic pair distribution function (mPDF): ground state of MnO. *Acta Crystallogr A Found Adv* **2015**, *71* (Pt 3), 325-334. DOI: 10.1107/S205327331500306X.

(18) Frandsen, B. A.; Yang, X.; Billinge, S. J. Magnetic pair distribution function analysis of local magnetic correlations. *Acta Crystallogr A Found Adv* **2014**, *70* (Pt 1), 3-11. DOI: 10.1107/S2053273313033081.

(19) Andersen, H. L.; Frandsen, B. A.; Gunnlaugsson, H. P.; Jorgensen, M. R. V.; Billinge, S. J. L.; Jensen, K. M. O.; Christensen, M. Local and long-range atomic/magnetic structure of non-stoichiometric spinel iron oxide nanocrystallites. *IUCrJ* **2021**, *8* (Pt 1), 33-45. DOI: 10.1107/S2052252520013585.

(20) Frandsen, B. A.; Gong, Z.; Terban, M. W.; Banerjee, S.; Chen, B.; Jin, C.; Feygenson, M.; Uemura, Y. J.; Billinge, S. J. L. Local atomic and magnetic structure of dilute magnetic semiconductor(Ba,K)(Zn,Mn)2As2. *Physical Review B* **2016**, *94* (9). DOI: 10.1103/PhysRevB.94.094102.

(21) Thomae, S. L. J.; Prinz, N.; Hartmann, T.; Teck, M.; Correll, S.; Zobel, M. Pushing data quality for laboratory pair distribution function experiments. *Rev Sci Instrum* **2019**, *90* (4), 043905. DOI: 10.1063/1.5093714.

(22) Juhás, P.; Davis, T.; Farrow, C. L.; Billinge, S. J. L. PDFgetX3: a rapid and highly automatable program for processing powder diffraction data into total scattering pair distribution functions. *Journal of Applied Crystallography* **2013**, *46* (2), 560-566. DOI: 10.1107/s0021889813005190.

(23) Billinge, S. J.; Farrow, C. L. Towards a robust ad hoc data correction approach that yields reliable atomic pair distribution functions from powder diffraction data. *J Phys Condens Matter* **2013**, *25* (45), 454202. DOI: 10.1088/0953-8984/25/45/454202.

(24) Greaves, C. A powder neutron diffraction investigation of vacancy ordering and covalence in γ-Fe2O3. *Journal of Solid State Chemistry* **1983**, *49* (3), 325-333. DOI: 10.1016/s0022-4596(83)80010-3.

(25) Wang, H. W.; Wesolowski, D. J.; Proffen, T. E.; Vlcek, L.; Wang, W.; Allard, L. F.; Kolesnikov, A. I.; Feygenson, M.; Anovitz, L. M.; Paul, R. L. Structure and stability of SnO2 nanocrystals and surface-bound water species. *J Am Chem Soc* **2013**, *135* (18), 6885-6895. DOI: 10.1021/ja312030e.

(26) Plekhanov, M. S.; Thomä, S. L. J.; Zobel, M.; Cuello, G. J.; Fischer, H. E.; Raskovalov, A. A.; Kuzmin, A. V. Correlating Proton Diffusion in Perovskite Triple-Conducting Oxides with Local and Defect Structure. *Chemistry of Materials* **2022**, *34* (10), 4785-4794. DOI: 10.1021/acs.chemmater.2c01159.

(27) Juhas, P.; Farrow, C. L.; Yang, X.; Knox, K. R.; Billinge, S. J. Complex modeling: a strategy and software program for combining multiple information sources to solve ill posed structure and nanostructure inverse problems. *Acta Crystallogr A Found Adv* **2015**, *71* (Pt 6), 562-568. DOI: 10.1107/S2053273315014473.

(28) Howell, R. C.; Proffen, T.; Conradson, S. D. Pair distribution function and structure factor of spherical particles. *Physical Review B* **2006**, *73* (9). DOI: 10.1103/PhysRevB.73.094107.

(29) Kodama, K.; Ikeda, K.; Shamoto, S.-i.; Otomo, T. Alternative Equation on Magnetic Pair Distribution Function for Quantitative Analysis. *Journal of the Physical Society of Japan* **2017**, *86* (12), 124708. DOI: 10.7566/jpsj.86.124708.

(30) Wright, J. P.; Bell, A. M. T.; Attfield, J. P. Variable temperature powder neutron diffraction study of the Verwey transition in magnetite Fe3O4. *Solid State Sciences* **2000**, *2* (8), 747-753. DOI: 10.1016/s1293-2558(00)01107-9.
